# Driving With Distraction: Measuring Brain Activity and Oculomotor Behavior Using fMRI and Eye-Tracking

**DOI:** 10.3389/fnhum.2021.659040

**Published:** 2021-08-16

**Authors:** Nicole H. Yuen, Fred Tam, Nathan W. Churchill, Tom A. Schweizer, Simon J. Graham

**Affiliations:** ^1^Department of Medical Biophysics, Faculty of Medicine, University of Toronto, Toronto, ON, Canada; ^2^Physical Sciences Platform, Sunnybrook Research Institute, Toronto, ON, Canada; ^3^Keenan Research Centre for Biomedical Science, St. Michael’s Hospital, Toronto, ON, Canada; ^4^Division of Neurosurgery, St. Michael’s Hospital, Toronto, ON, Canada

**Keywords:** driving simulation, distraction, neural correlates of driving, fMRI, eye-tracking

## Abstract

**Introduction:**

Driving motor vehicles is a complex task that depends heavily on how visual stimuli are received and subsequently processed by the brain. The potential impact of distraction on driving performance is well known and poses a safety concern – especially for individuals with cognitive impairments who may be clinically unfit to drive. The present study is the first to combine functional magnetic resonance imaging (fMRI) and eye-tracking during simulated driving with distraction, providing oculomotor metrics to enhance scientific understanding of the brain activity that supports driving performance.

**Materials and Methods:**

As initial work, twelve healthy young, right-handed participants performed turns ranging in complexity, including simple right and left turns without oncoming traffic, and left turns with oncoming traffic. Distraction was introduced as an auditory task during straight driving, and during left turns with oncoming traffic. Eye-tracking data were recorded during fMRI to characterize fixations, saccades, pupil diameter and blink rate.

**Results:**

Brain activation maps for right turns, left turns without oncoming traffic, left turns with oncoming traffic, and the distraction conditions were largely consistent with previous literature reporting the neural correlates of simulated driving. When the effects of distraction were evaluated for left turns with oncoming traffic, increased activation was observed in areas involved in executive function (e.g., middle and inferior frontal gyri) as well as decreased activation in the posterior brain (e.g., middle and superior occipital gyri). Whereas driving performance remained mostly unchanged (e.g., turn speed, time to turn, collisions), the oculomotor measures showed that distraction resulted in more consistent gaze at oncoming traffic in a small area of the visual scene; less time spent gazing at off-road targets (e.g., speedometer, rear-view mirror); more time spent performing saccadic eye movements; and decreased blink rate.

**Conclusion:**

Oculomotor behavior modulated with driving task complexity and distraction in a manner consistent with the brain activation features revealed by fMRI. The results suggest that eye-tracking technology should be included in future fMRI studies of simulated driving behavior in targeted populations, such as the elderly and individuals with cognitive complaints – ultimately toward developing better technology to assess and enhance fitness to drive.

## Introduction

Distracted driving poses a danger to drivers, passengers, pedestrians and cyclists and is a growing threat to road safety across the world. For example, the United States (US) National Highway Traffic Safety Administration reported 37,461 fatalities caused by motor vehicle crashes (MVCs) in 2016, with over 2 million people in the United States injured in MVCs each year (2018). In many of these cases, the MVC occurred because drivers were engaged in dangerous multi-tasking (including activities such as texting, eating, or talking to a passenger). Such behavior can distract the driver from performing driving-related tasks safely, such as maintaining proper lane position (Hamish Jamson and Merat, 2005). The rapid development and widespread use of smart cellphone technology in particular has increased concerns over distracted driving - as texting while driving takes a hand away from the wheel, decreases visual attention to the road (Fitch et al., 2015), and impairs driving response and reaction time (Strayer and Drew, 2004; Drews et al., 2009). Studies have shown that distracted driving with cell phone usage results in failure to stop completely at stop signs, delayed braking responses and more rear-end collisions (Strayer and Drew, 2004; Kramer et al., 2006; Thompson et al., 2012). Cell phone usage alone has been attributed to approximately 15 – 25% of fatal distracted driving crashes (Ortiz et al., 2016).

From the perspective of psychological science, driving is a complex, demanding task that requires numerous cognitive abilities, including attention, memory, decision-making, and alertness to adapt to a rapidly changing environment. A decline in driving performance can arise due to processes that negatively affect such abilities, such as distraction (Lee et al., 2003; Cantin et al., 2009; Rizzo, 2011). Distraction, introduced as a secondary task that requires executive functions (as opposed to distraction by sudden, attention capturing events, such as children running into the street) has the potential to divert attention and resources from the primary driving task, thereby increasing risk of MVCs from degraded driving ability (Thompson et al., 2012).

Moreover, cognitive abilities are relevant for processing and responding to the continuous stream of sensory input that is received by the driver, requiring them to make informed and correct decisions, especially during complex road or traffic scenarios. Examples of these scenarios include driving at busy intersections, making left turns with oncoming traffic, or merging into traffic and making lane changes (Thompson et al., 2012; Noyce et al., 2017). Declines in cognitive abilities also pose a safety risk and potentially make the driver more prone to the effects of distracted driving (Fraade-Blanar et al., 2018). Elderly and middle-aged drivers have been shown to commit significantly more driving errors and reduced steering wheel control when distracted (Thompson et al., 2012).

In many cases, physicians lack quantitative and objective tools to assess fitness to drive in patients, and must make such decisions based on clinical exam and rapid cognitive assessments that are often insufficiently informative (Marshall and Gilbert, 1999; Withaar et al., 2000). The decision to declare an individual unfit to drive is also complicated by the fact that in the developed world, driving is very often key to functional dependence and to employment.

Neuropsychological tests (NPTs) may also have a role in clinical and scientific settings in the assessment of fitness to drive and the ability to drive safely. These standardized behavioral tests are more detailed than clinical assessments and can be used to assess cognitive functions that underpin driving abilities, determining whether test performance is impaired in relation to population norms (Lundberg et al., 1997; Lezak et al., 2004). Although NPTs are useful to identify cognitive decline, they have not been established as good predictors of driving ability, however. For example, one study investigating a range of NPTs found that the best four-test combination was able to identify at-risk drivers with 95% specificity, but only 80% sensitivity (Bowers et al., 2013). Further research is needed to develop complex cognitive tasks or screening tools to predict driving performance better. As an initial part of this work, there is a strong need to understand the underlying brain activity associated with normal and unimpaired driving performance. From this information, it may be possible in the future to develop objective, robust behavioral methods to assess fitness to drive.

Many researchers have followed this line of thinking to study the task-related brain activity associated with various types of driving behavior, especially using the method of functional magnetic resonance imaging (fMRI). By necessity, these studies involve simulated rather than actual driving behavior, using various forms of virtual reality or video game technology to give test participants the simulated experience of driving while they lie in the magnet bore of an MRI system. The costs associated with fMRI and other practical considerations (e.g., access and availability) make it very unlikely that this imaging method can provide direct utility as a clinical tool for assessing fitness to drive – however, fMRI is very useful for providing scientific insight. Simulated driving studies using fMRI have revealed a broad network of task-related brain activity, engaging areas in all major lobes of the brain (Calhoun et al., 2002; Graydon et al., 2004; Callan et al., 2009; Crundall and Underwood, 2011; Calhoun and Pearlson, 2012; Choi et al., 2017). In simple straight driving, increased activations in the visual-association, parietal and occipital regions were observed, related to visuomotor integration; additionally, the precentral gyrus, superior and inferior parietal lobules, and cerebellum were seen to activate and contribute to motor control (Calhoun and Pearlson, 2012). In a study investigating driving while concurrently performing arithmetic tasks, activations of the motor cortex, parietal and occipital lobes were seen to decrease in comparison to the driving only task; whereas the temporal and inferior frontal regions, associated with auditory processing and additional task performance, showed an increase in activation when concurrently performing a secondary task (Choi et al., 2017). Similarly, activation in the motor cortex, parietal and occipital lobes and superior temporal gyrus was reported when driving with an auditory task (Uchiyama et al., 2012). Furthermore, an auditory distraction task was reported to shift activation from the posterior and visual areas to the frontal regions, which are responsible for planning, decision-making and cognition (Schweizer et al., 2013).

Despite the knowledge gained from these studies, the interpretation of the brain activity associated with simulated driving behavior remains limited, as many brain areas are active when performing simulated driving tasks and it is difficult to link the contribution of specific areas to the various behavioral subcomponents of driving behavior. One method to overcome this difficulty involves augmenting the behavioral recording that is undertaken during fMRI experiments of simulated driving. In particular, vision and visual attention are extremely crucial to safe driving. Attention has been described to be involved in guiding eye movements, including point of gaze spatiotemporal characteristics, eye blink rate and pupil dilation (Schneider, 1995; Moore and Fallah, 2001). Such parameters can be readily measured using modern eye-tracking devices (Land, 2006). The introduction of a secondary task to driving has been shown to decrease the percentage of gaze points in peripheral regions (e.g., speedometer, rearview mirror), and to cause spatial gaze concentration due to the increased mental workload (Recarte and Nunes, 2003; Tsai et al., 2007). In addition, eye blink rate and duration have been shown to have associations with cognitive load in a range of behavior including word-naming tasks (Ohira, 1996), visuospatial memory tasks (Van Orden et al., 2001) and driving in various environments (Brookings et al., 1996; Tsai et al., 2007; Haak et al., 2009; McIntire et al., 2014; Faure et al., 2016). It has been suggested that the blink rate decreases to minimize the loss of incoming information, or to prevent disruption to cognitive processes involved in the mental task (Holland and Tarlow, 1972; McIntire et al., 2014; Maffei and Angrilli, 2018). One study that investigated changes in driving behavior in the presence of a secondary cognitive task found that when the driving environment was more demanding, blink rate decreased (Faure et al., 2016). Pupil dilation has also been observed to modulate cognitive processing. Greater dilation has been correlated with greater cognitive load across various mental tasks (Recarte and Nunes, 2003; Tsai et al., 2007; Palinko et al., 2010; Gable et al., 2015; Niezgoda et al., 2015) and individuals with better task performance have been shown to undergo a larger percent change in pupil size (Tsai et al., 2007; Palinko et al., 2010).

Because eye-tracking technology can be used to measure cognitive load, combining eye-tracking with fMRI is likely to support and inform the interpretations of the ensuing activation maps associated with simulated driving behavior by providing additional information about the participant behaviors during each task. At present, no studies have been conducted using both fMRI and eye-tracking simultaneously in the context of simulated driving.

To fill this gap in the scientific literature, the present study adopts simultaneous eye-tracking and fMRI. It is hypothesized that complex driving conditions will engage greater brain activity in areas involved in visual and motor processing, and that the addition of a distracting cognitive task will engage medial and lateral frontal areas, while decreasing the extent of occipital activation. It is also hypothesized that an increase in driving task demand and distraction during simulated driving will result in decreased gaze distribution and blink rate, and increased pupil diameter, and that these effects will correlate with similar changes in fMRI signals.

## Materials and Methods

### Ethics Statement

This study was carried out in accordance with the recommendations of the Research Ethics Board at Sunnybrook Health Sciences Centre, Canada. All participants were given a full explanation of the experimental procedures and provided written informed consent prior to participating in the study.

### Participants

Nineteen healthy adults between the ages of 20 and 30 were recruited for this study. Seven participants were excluded due to unusable eye-tracker data quality (arising from factors such as blue eye color, and obstruction of the view between the eye and the eye-tracking video camera due to long eyelashes). Twelve participants remained for subsequent analysis (4 females and 8 males, mean age = 23.4 years, SD = 1.1 years) with mean driving experience of 5.3 years (SD = 2.1 years, range = 1.5 – 9.0 years). All participants had a valid driver’s license in the Canadian province of Ontario, no history of psychological or neurological illness, and were able to be imaged in a 3 T MRI system (i.e., they were free from MRI exclusion criteria, such as claustrophobia or ferromagnetic implants). All participants were right-handed with normal or corrected vision. Ten of the participants were right-eye dominant (4 females, 6 males), whereas two were left-eye dominant (2 males).

### Driving Simulation

Simulated driving tasks were administered using STISIM Drive Software (Systems Technology, Inc., Hawthorne, CA, United States), controlled by custom fMRI-compatible driving simulator hardware (with foot pedals and steering wheel) (Kan et al., 2012). The steering wheel included response buttons embedded on the wheel that enabled participants to answer questions while driving, similar to modern steering wheels in real life that include buttons to adjust volume or answer cell phone calls. The simulation environment was shown on a screen using an fMRI-compatible projector (Avotec SV-6011 LCD Projection System, Stuart, FL). The environment was observed by the participants through a mirror mounted on the head coil, and auditory stimuli were delivered through fMRI-compatible headphones (Avotec SS-3100, Stuart, FL).

Prior to the fMRI experiment, participants underwent training in an fMRI simulator for approximately one hour to familiarize them with the driving hardware and software. The training scenario included the same tasks to be performed during fMRI, in pseudo-randomized order. Participants were told to follow traffic laws and maintain the posted speed limit of 60 km/h during the session.

#### Driving Tasks

**FIGURE 1:**
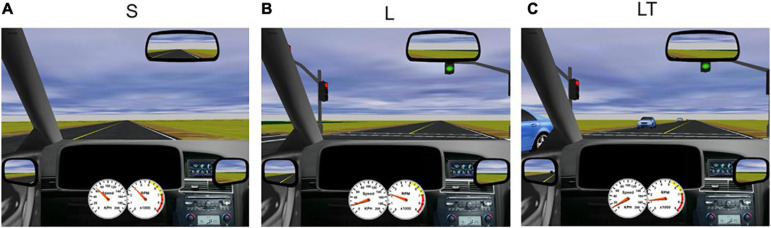
Screenshots of STISIM driving scenarios. **(A)** S, straight driving; **(B)** L, left turn at an intersection without traffic; and **(C)** LT, left turn with traffic.

When in the magnet, participants were again instructed to adhere to traffic lights and road rules while maintaining the posted speed of 60 km/h. The environment of the driving scenario was mainly rural, with minimal scenery, trees and buildings. Driving tasks included straight driving (“Straight,” “S,” Figure 1A) and turns at intersections with and without oncoming traffic, with or without having to perform an auditory task simultaneously. At task onset, participants were presented with an auditory recording of a male voice to mimic instructions from a modern navigation device (e.g., “At the intersection, turn right”). The recording occurred one hundred virtual meters in advance of the intersection (a time duration of approximately 6 s when driving at the speed limit). For all turning tasks, the intersection included a set of traffic lights with the appropriate light in the green “go” condition. Each participant performed eight trials of right-hand turns (“Right Turn,” “R,” not shown) and seven trials of left-hand turns (“Left Turn,” “L,” Figure 1B) without any traffic or distraction. To increase complexity of the driving task, oncoming traffic was introduced to left turns (without distraction), which required participants to decide when it was safe to turn (“Left Turn + Traffic,” “LT”; seven trials, Figure 1C). To simulate distraction, an auditory task was presented to the participant as a general knowledge true or false question (e.g., “a hammer is lighter than a feather”) during straight driving (“Straight + Audio,” “SA”; six trials) and in left turns with oncoming traffic (“Left Turn + Traffic + Audio,” “LTA”; seven trials), immediately after the turn instruction was presented. Questions were answered by pressing buttons corresponding to true and false on the steering wheel. During LTA and SA conditions, participants were not told to prioritize either the driving or the auditory task; and no error feedback was provided throughout all the task conditions, other than what each participant detected using their senses. The simulated driving scenarios were administered in pseudo-randomized order and were split into two runs to enable participants to remain vigilant without becoming fatigued. Each trial duration was approximately 20 s long, and each run had a duration of approximately 12 min (depending on the driving speed of the participant), for a total driving time of approximately 25 min. Straight driving after each turn task served as the baseline condition and was the same for all participants. Driving task trials were separated by at least 10 s of baseline straight driving to minimize overlapping of fMRI and eye movement response signals between the various driving task conditions.

### Functional MRI Protocol

Once each participant completed the fMRI simulator session, they were asked to complete a screening form to ensure that they were able to undergo fMRI. The form was reviewed by the MRI technologist and after clearance was granted to enter the magnet room, the participant was positioned on the patient table of the MRI system. To increase participant comfort and to minimize body and head motion, pillows were placed under the knees for leg support, and pads were placed under the arms and elbows and beside the head. The foot pedals (accelerator and brake) were placed appropriately such that when they were fully depressed, there was no bulk body movement that could translate into unwanted head motion. The steering wheel was then placed over the torso. The fMRI-compatible headset was fitted on the participant over noise-reducing ear plugs, and the sound system was tested to ensure that the participant was able to hear instructions clearly during the driving scenarios. Because both hands were used to steer, the peripheral pulse monitor was placed on the toe to record heart beat information, rather than the standard placement on the finger. Respiration was monitored, and an emergency call squeeze bulb was placed on the participant so that they could discontinue the study for any reason, if they wished.

Two fMRI runs with simulated driving were performed to enable the participant to maintain vigilance throughout task performance and to reduce fatigue. Anatomical imaging was acquired at high spatial resolution in between the runs to allow the participant to rest their eyes. The fMRI session took approximately one hour to complete, involving 30 min of setup and calibration and 30 minutes of imaging. The driving scenarios were triggered synchronously with the start of fMRI data collection. Images were acquired using a 3.0 Tesla MRI system (Prisma, Siemens, Erlangen, Germany). Functional MRI during simulated driving was performed using T2^∗^-weighted echo planar imaging (EPI; repetition time (TR) = 1.75 s, echo time (TE) = 30 ms, flip angle (FA) = 40°, field of view (FOV) = 256 × 256 mm^2^, 60 slices, voxel size = 2.5 × 2.5 × 2.5 mm^3^, 377 time points). Anatomical imaging was performed using T1-weighted, three-dimensional magnetization prepared rapid acquisition gradient echo imaging (3D MPRAGE; TR = 1.8 s, TE = 2.21 ms, FA = 10°, FOV = 256 × 256 mm^2^, 176 slices, voxel size = 1.0 × 1.0 × 1.0 mm^3^).

### Eye-Tracking Setup

An fMRI-compatible high-speed eye-tracker was used to record movements of the right eye for consistency of methodology, at 1000 Hz using a monocular system with a 50 mm lens (EyeLink 1000 Plus, SR Research Ltd., Mississauga, Canada) and infrared illumination (910 nm wavelength). The eye was tracked using corneal reflection, and the pupil was detected using a centroid fitting algorithm, using the standard hardware and software of the system. Metrics that were measured included brief pauses (ocular fixations) as well as rapid shifts (saccades) in the point of gaze, fixations and blinks. Each sample included the time latency (measured from the time the tracker software was started), eye position on the display screen (measured as coordinates on the calibrated display screen), and pupil size (measured in arbitrary units).

If vision correction was required, the appropriate prescription MRI-compatible lens was selected and prepared. When the lens was placed in MRI-compatible frames, it created reflection onto the eye-tracker camera, preventing the eye to be tracked. To avoid this, the lens was gently placed on the participant’s face, supported with tape and cotton pads (five participants). To calibrate the eye-tracking system, the participant was instructed to fixate on each of thirteen target locations on the display screen, appearing consecutively after a fixation on the target was detected. Following calibration, a validation procedure was undertaken to measure gaze position accuracy on the screen using the prior calibration parameters. Calibration and validation were performed before each simulated driving run. Eye-tracking measurements were started manually, then subsequent initialization of the fMRI acquisition sent a synchronous trigger to the eye-tracker to denote the time that the fMRI and driving scenario were started.

### Data Extraction and Analysis

#### Driving Simulator Data

Driving simulator data were automatically generated and saved at the end of each driving scenario as an STISIM Drive data file. This output was parsed to extract the time series data of driving metrics that were measured. Driving metrics extracted included length of run, longitudinal position, lane position (deviation of lateral lane position of the vehicle referenced in relation to the center of the driver vehicle with respect to the roadway center dividing line), driving speed, vehicle heading angle, and number of collisions. These metrics, which have been used to characterize impaired driving performance in various patient populations with neurological impairment (e.g., Mansur et al., 2018; Hird et al., 2017), were converted into a matrix of size N × M (with N variables and M time points) and read into MATLAB (The MathWorks Inc., Natick, MA, United States). Metrics that were calculated in MATLAB included event onset time (longitudinal position of event onset converted into time), time to turn (time between event onset and when the vehicle heading angle was at 25°, considered to be start of the turn position), turn speed (mean speed between turn start time and turn end time, after the vehicle heading angle returned to 0°), and question response time (time a response button was pressed minus the time the question was administered). Metrics of interest were concatenated for both driving runs. Statistical analyses of behavioral data were completed using Statistical Product and Service Solutions (SPSS) software (IBM SPSS Statistics Version 25, IBM, Armonk, NY, United States). Repeated Measures ANOVA tests were used to determine if there were significant differences between the time to turn and turn speed across R, L, LT, and LTA conditions, with subsequent Bonferroni-corrected *post hoc* contrasts to determine which factors were significantly different. Paired samples *t*-tests were used to assess significance of driving speed and lane position between S and SA conditions, and question response time and accuracy between SA and LTA conditions.

#### MRI Data

The fMRI time series data were preprocessed using Analysis of Functional NeuroImages (AFNI) freeware (Cox, 1996) to remove large spikes in the time series, to correct for the physiological effects of the cardiac and respiratory cycles, to suppress artifacts from head motion correction (and ensuring no excessive head motion), and to correct for the slight differences in time that each image slice was collected in each multi-slice acquisition of the brain volume (i.e., “slice-time correction”). The data were spatially smoothed using a 5 mm full width at half maximum Gaussian kernel, then normalized by the mean of each voxel. From the driving simulator data, the times of each event onset for all trials of all tasks (L, R, LT, LTA, S and SA conditions as defined in section “Driving Tasks”) were extracted for both driving runs and saved as individual text files for each task. These event onset times, reflecting the time the auditory instructions were presented, were used to enter a stimulus timing file into a general linear model (GLM) using straight driving (the S condition) as the control, to estimate task-related brain activity at each voxel location. The stimulus-timing file, which included the full 20 s duration of each driving task, was convolved with a canonical hemodynamic response function with six regressors (head motion parameters in six degrees of freedom). The maps were then averaged in Talairach and Tournoux (TT) atlas space (Talairach and Tournoux, 1988) and thresholded using a false discovery rate (Genovese et al., 2002) of q = 0.05 to correct for multiple statistical comparisons. Maps for all 12 participants were entered into a within-participants, random effects analysis of variance (ANOVA) to create final group activation maps, which were overlaid on an anatomical average in Talairach atlas space for interpretation. Activation maps were generated for the following task contrasts of interest: R - S, L - S, (L – S) – (R – S), LT - S, LT – L, LT – R, (LT – L) – (LT – R), SA - S, LTA - S, LTA - SA and LTA - LT.

#### Eye-Tracker Data

The eye-tracker output data were converted from the native Eyelink Data File (.EDF) to ASCII text format and were then read into MATLAB. These data consisted of the raw pupil size time series, blink start and end times, saccade start and end times, fixation start and end times and the associated gaze locations on the display screen. The first data pre-processing step involved removing invalid pupil size samples (non-positive values, created by loss of eye target, eyelid occlusion or blinks). The remaining pupil size samples were subjected to additional criteria for removal, including dilation speed outliers and edge artifacts, trend-line deviation outliers, and samples that were temporally isolated (Kret and Sjak-Shie, 2019). Dilation speed outliers are samples that have a disproportionately large absolute pupil size change relative to adjacent samples, likely due to artifacts. These were detected based on the median absolute deviation calculated from the dilation speed. Trend-line deviation outliers are samples that deviate greatly from the signal trend line, which was generated through interpolation and data smoothing. Temporally isolated samples are likely the result of noise or system error (e.g., pupil detection when eyes are shut or not properly tracked) (Kret and Sjak-Shie, 2019).

A temporal threshold of 100 ms was subsequently used in the eye-tracking data to distinguish between fixations and saccades. This threshold is suitable for tasks that involve visual processing of complex geometric stimuli, whereas highly automatic processing (e.g., of the human face) typically involves longer fixations (Manor and Gordon, 2003). Fixations were grouped by tasks, then further grouped by areas of interest (road, non-road) within each task. Percentage of time spent in fixation (i.e., percent fixation duration) was measured as the total duration of fixations from the time of trial onset to the time the participant turned, divided by this time duration and multiplied by 100%. The spatial distribution of percent fixation duration was determined for each participant by the additive superposition of Gaussians, centered at each fixation location and weighted by the fixation duration. The distribution maps for each of the L, LT and LTA conditions were then averaged over all participants, and depicted as colored contour lines overlaid on the driving simulation screen. A Gaussian function was also fit to the distributions for each participant, resulting in estimated values for the percent fixation duration peak and width for each task condition. The sampling distributions of the peaks and widths were tested for normality using the Kolmogorov-Smirnov test using SPSS, then tested for differences using paired samples t-tests and 95% Confidence Interval (CI) testing by bootstrapping.

Saccades that did not meet the minimal saccadic duration of 10 ms were removed, and the percent saccade duration was measured as the total duration of saccades over the length of the trial (Nij Bijvank et al., 2018). The saccade amplitude, peak velocity, and number of performed saccades were also compared for each task.

The average percentage duration of each eye behavior, including saccades, blinks and fixations, was also plotted on a stacked bar graph for each left turn condition from the time of trial onset to the time of turning. Fixations were categorized as “road” (including motor vehicles), and non-road (including the horizon and interior locations of the driven car, such as the rear-view mirror and speedometer). In separate analyses, the effect of blink rate and pupil size was investigated over the trial duration, measured as the time of trial onset to offset. Blink rate was calculated for each task as the number of recorded blinks for each participant divided by the total trial duration (reported as blinks per minute). Similarly, the mean pupil dilation size was calculated as the temporally averaged pupil size for each task condition.

#### Relationships Between BOLD Signal and Eye-Tracking Metrics

To investigate the relationships between the BOLD signal and eye-tracking metrics, the mean BOLD signal intensity was first calculated in key brain regions that have been associated with eye movements, attention and cognitive processing (inferior frontal, inferior occipital, medial frontal, middle frontal, middle occipital, orbitofrontal, superior frontal, superior occipital, superior temporal and thalamic regions). The brain regions of interest were masked and the time series were averaged over all voxels in a given mask region. The mean BOLD signal intensity was then calculated for each left turn task (L, LT, and LTA). The differences in BOLD signal from the LT condition were then calculated as a change score for each region of interest (i.e., mean BOLD signal intensity of L – mean BOLD signal intensity of LT, and mean BOLD signal intensity of LTA – mean BOLD signal intensity of LT). Change scores for blink rate and pupil dilation were calculated in the analogous manner. Scatter plots were then generated to compare the change scores for the BOLD signal intensity and each eye-tracking metric to investigate the relationships based on the Pearson correlation value.

## Results

### Driving Behavior

**FIGURE 2:**
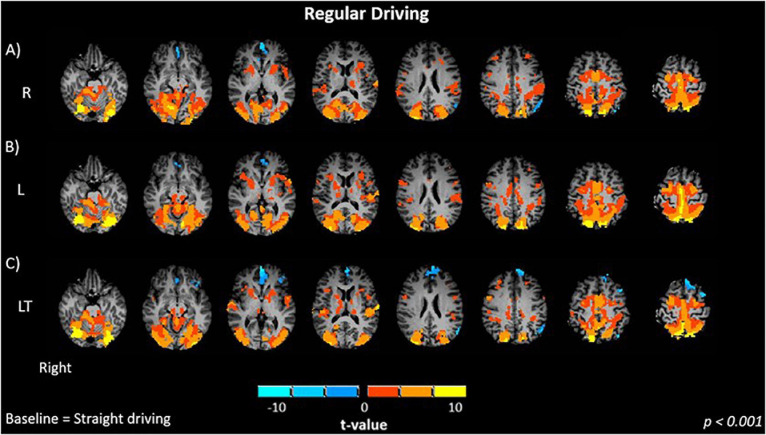
Brain activations of participants when performing regular driving tasks: **(A)** R, right turns; **(B)** L, left turns; **(C)** LT, left turns with oncoming traffic. Activations are shown as *t* contrasts in comparison with straight driving, and are reported with a statistical significance threshold of *p* < 0.001. “Right” indicates the right cerebral hemisphere.

Descriptive statistics for the behavioral metrics of simulated driving performance, as obtained from the STISIM software and further calculated in MATLAB, are shown in Table 1 for the S, SA, R, L, LT, and LTA conditions. Participants followed the rules of the road and were able to complete all the driving conditions “safely”: a total of 2 collisions was observed across all participants for each of R, LT and LTA conditions, whereas only 1 collision was observed for the L condition (The metric was not relevant for the S and SA conditions). Moreover, a number of the behavioral metrics were not significantly different across the driving conditions. This included the time to turn (Repeated Measures ANOVA; *p* > 0.05), and lane position for the S and SA conditions (paired samples *t*-test; *p* > 0.05). However, a paired samples t-test showed that driving speed (reported as the mean (SD), where SD is the standard deviation) for the SA condition (60.4 (0.8) km/hr) was significantly slower than that of S condition (62.5 (2.0) km/hr). In addition, there was a significant effect of turning condition on turn speed (Repeated Measures ANOVA; *p* < 0.05; Figure 2B). Bonferroni-corrected *post hoc* contrasts showed no significant differences in the turn speed between L and LT conditions, and between R and LTA conditions. The turn speed was significantly slower for the R condition (30.5 (5.9) km/hr) than for the L condition (37.9 (4.8) km/hr), and similar contrast was found between the R and LT condition (35.6 (3.4) km/hr) (corrected *p* < 0.05). In addition, the turn speed for the LTA condition (34.3 (3.1) km/hr) was significantly slower than for the L condition (corrected *p* < 0.05).

**TABLE 1:** Descriptive statistics of behavioral metrics of simulated driving performance: turn speed, time to turn and number of collisions in the turning tasks (R, L, LT, and LTA), driving speed and lane position in straight driving tasks (S, SA), and response time and response accuracy in audio tasks (SA, LTA).

**Condition**	**Turn speed**				**Time to turn**				**Collisions**	
	**Mean (km/hr)**	**SD (km/hr)**	**95% CI**		**Mean** **(s)**	**SD** **(s)**	**95% CI**		**total count**	
			**LB**	**UB**			**LB**	**UB**		
R	30.5	5.9	26.8	34.3	10.4	1.3	9.6	11.3	2	
L	37.9	4.8	34.9	40.9	10.2	1.2	9.4	11.0	1	
LT	35.6	3.4	33.4	37.7	9.9	2.5	8.3	11.5	2	
LTA	34.3	3.0	32.4	36.3	10.5	1.7	9.4	11.6	2	
	**Driving speed**						**Lane position**			
		**Mean (km/hr)**		**SD (km/hr)**	**95% CI**		**Mean (m)**	**SD** **(m)**	**95% CI**	
					**LB**	**UB**			**LB**	**UB**
S		62.5		2.0	61.2	63.8	2.2	0.4	1.9	2.5
SA		60.4		0.8	59.9	60.9	2.3	0.3	2.1	2.5
	**Response time**						**Response accuracy**			
		**Mean (s)**		**SD** **(s)**	**95% CI**		**Mean (%)**	**SD (%)**	**95% CI**	
					**LB**	**UB**			**LB**	**UB**
SA		4.1		0.7	3.6	4.6	83.3	20.1	70.6	96.1
LTA		4.1		1.3	3.3	5.0	77.4	14.2	68.3	86.4

*SD, standard deviation; CI, Confidence Interval; LB, Lower Bound; UB, Upper Bound. R, right turns; L, left turns; LT, left turns with oncoming traffic; LTA, left turns with oncoming traffic and auditory distraction; S, straight driving; SA, straight driving with auditory distraction.*

Regarding the auditory task performance during distracted simulated driving, paired samples t-tests did not reveal significant differences between the SA and the LTA conditions, neither for question response time, nor response accuracy (*p* > 0.05). Notably, the mean (SD) of the response time was 4.1 (1.0) s over both conditions, whereas the time to turn in the LTA task was 10.5 (1.7) s. Because the time to turn was not significantly elevated for the LTA condition in comparison to the other turn conditions and the turn speed was slower for the LTA condition than for the L condition, this suggests that participants were dual-tasking rather than performing the two tasks sequentially during the LTA condition. If the tasks were performed sequentially, then a time to turn of approximately 10.5 + 4.1 = 14.6 s would have been expected for the LTA task. As this was not observed, it can be inferred that the tasks were performed in parallel.

### Brain Activity

#### Regular Driving

Brain activations are reported relative to the S control condition. The R (Figure 2A) and L (Figure 2B) turning conditions without traffic showed similar activation maps (*p* < 0.001), and contrast maps between the two conditions were not significantly different. Both R and L conditions showed significant positive bilateral activation in the occipital (lingual gyrus, middle occipital), inferior parietal, primary motor, primary somatosensory and premotor cortices. Activation was also observed in the precuneus, cerebellum and basal ganglia.

The addition of oncoming traffic to left turns (LT, Figure 2C) produced activation maps (*p* < 0.001) that were very similar to those for the R and L conditions. Although negative activations were slightly more extensive in the frontal (left inferior, left and right medial, left middle, left superior) cortex and the anterior cingulate cortex, the contrast maps between the regular driving conditions (LT – L, LT – R) did not show statistically significant differences (*p* > 0.01).

#### Distracted Driving

**FIGURE 3:**
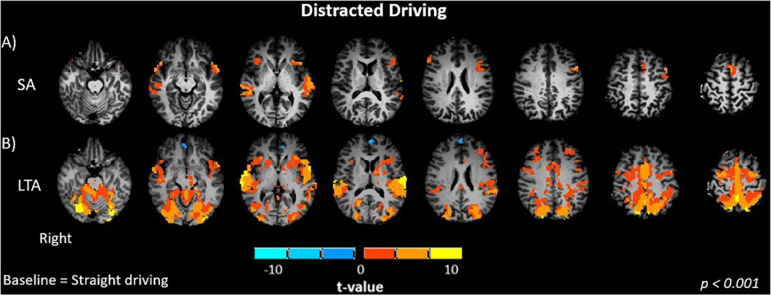
Brain activations of participants when performing driving tasks while distracted. **(A)** SA, straight driving with audio task; **(B)** LTA, left turns with oncoming traffic and audio. Activations are shown as t contrasts in comparison with straight driving, and are reported with a statistical significance threshold of *p* < 0.001. “Right” indicates the right cerebral hemisphere.

When evaluating the differences in brain activation between distracted straight driving and straight driving conditions, the (SA – S) contrast showed significant positive activations in the temporal (left and right superior, right middle) and frontal (left and right inferior) cortices and the anterior cingulate cortex (*p* < 0.001, Figure 3A). No significant activation was observed in the occipital, parietal and somatosensory areas. The (LTA – S) contrast was evaluated in analogous fashion. In addition to activations in occipital, parietal, motor, somatosensory and cerebellar regions observed for the L and LT conditions, the LTA condition showed greater positive activations in relation to the S condition in frontal (right middle, left and right inferior) and temporal (left and right middle and superior) cortices, anterior cingulate cortex and insula (*p* < 0.001, Figure 3B).

**FIGURE 4:**
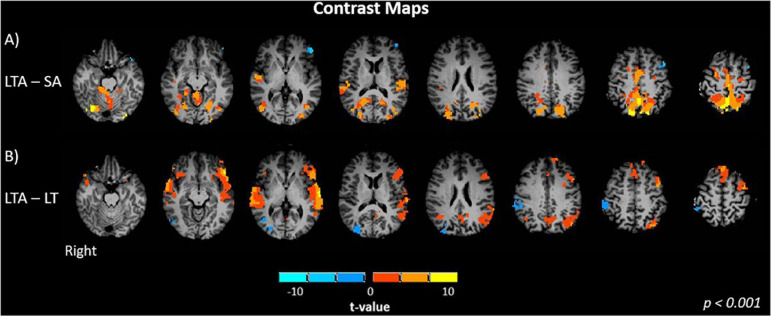
Brain activation maps of contrast between task factors: **(A)** LTA – SA, Left turns with oncoming traffic and audio vs. straight driving with audio; and **(B)** LTA – L, left turns with oncoming traffic and audio vs. left turns with oncoming traffic without audio. Activations are shown with a statistical significance threshold of *p* < 0.001. “Right” indicates the right cerebral hemisphere.

Next, the difference maps between LTA – SA and LTA – LT are shown in Figure 4. These two contrasts are of interest as they serve to illustrate the differing effects of distraction response for different driving demands. Compared to SA, LTA showed significantly greater activation in the left and right superior temporal gyrus (*p* < 0.001, Figure 4A). Significant increases in activation were also seen in the occipital (middle, superior); the primary motor, somatosensory and premotor cortices; as well as the precuneus and cerebellum. Significant decreases in activation were seen in the left inferior frontal gyrus. Compared to LT, LTA showed significantly greater activation in the frontal (left and right inferior, left superior, left medial, left middle), temporal (left and right middle, left and right superior) cortices and precuneus (*p* < 0.001, Figure 4B). Significant decreases were observed in the right occipital (middle, superior), right inferior parietal, and cerebellar areas.

The activations shown in Figures 2–4 are subsequently listed in Table 2, which provides the *t*-value of peak activation, the location of each peak in Talairach and Tourneaux (TT) atlas coordinates and the associated brain region in the TT atlas.

**TABLE 2:** Peak activation (*t*-value), spatial coordinates (Left, Posterior, Inferior) and brain region of statistically significant brain activity with respect to the Talairach and Tournoux (TT) brain atlas.

**Peak activation intensity (*t*-value)**	**Atlas coordinates**			**TT Atlas region**
	***L***	***P***	***I***	
**R - S**				
16.45	9	59	64	Left Precuneus, BA 7
14.20	11	34	71	Left Postcentral Gyrus, BA 3
9.89	−26	61	−14	Right Declive
7.48	39	64	−14	Left Fusiform Gyrus, BA 19
7.06	29	66	−14	Left Declive
7.03	−1	1	49	Right Medial Frontal Gyrus, BA 6
6.39	−11	69	6	Right Cuneus, BA 18
6.04	−11	76	4	Right Lingual Gyrus, BA 18
5.93	−21	6	61	Right Middle Frontal Gyrus, BA 6
5.03	9	69	11	Left Cuneus, BA 30
5.02	9	94	-9	Left Lingual Gyrus, BA 17
**L - S**				
12.67	−14	79	41	Right Precuneus, BA 7
10.10	26	74	−16	Left Declive
9.72	−16	81	36	Right Cuneus, BA 19
9.51	−24	66	−14	Right Declive
8.14	−1	14	54	Right Medial Frontal Gyrus, BA 6
7.86	1	26	56	Left Medial Frontal Gyrus, BA 6
6.21	26	49	−11	Left Culmen, BA 37
6.11	29	84	6	Left Middle Occipital Gyrus, BA 18
5.94	−19	59	16	Right Posterior Cingulate, BA 30
5.85	−24	49	−11	Right Culmen, BA 37
5.75	−1	41	−9	Right Cerebellar Lingual
**LT - S**				
11.38	−14	79	41	Right Precuneus, BA 7
9.51	−26	66	−16	Right Declive
8.60	26	76	−16	Left Declive
6.62	−4	1	46	Right Cingulate Gyrus, BA 24
6.34	−1	11	49	Right Medial Frontal Gyrus
6.08	1	76	−1	Left Lingual Gyrus, BA 18
6.06	6	44	49	Left Precuneus, BA 7
5.82	16	36	−39	Left Cerebellar Tonsil
5.66	−24	46	−11	Right Culmen, BA 37
5.38	44	26	11	Left Transverse Temporal Gyrus, BA 41
5.07	−11	46	−46	Right Cerebellar Tonsil
5.02	−19	59	19	Right Posterior Cingulate
**SA - S**				
4.48	1	−9	51	Left Superior Frontal Gyrus, BA 6
4.33	−49	31	1	Right Middle Temporal Gyrus, BA 21
3.71	−49	39	6	Right Superior Temporal Gyrus, BA 22
**LTA - S**				
9.39	−29	66	−16	Right Declive
8.28	−1	56	49	Right Precuneus, BA 7
7.13	16	79	36	Left Precuneus, BA 7
7.05	29	66	−14	Left Declive
6.92	−51	21	11	Right Transverse Temporal Gyrus, BA 41
6.47	−1	6	44	Right Cingulate Gyrus, BA 24
6.32	−1	11	51	Right Medial Frontal Gyrus, BA 6
5.36	1	-11	39	Left Cingulate Gyrus, BA 24, 32
**LTA - SA**				
10.18	−1	56	49	Right Precuneus, BA 7
7.67	−29	69	−14	Right Declive
6.60	−19	56	19	Right Posterior Cingulate
6.46	−21	26	−21	Right Culmen
5.91	−1	11	51	Right Medial Frontal Gyrus, BA 6
5.53	46	24	11	Left Transverse Temporal Gyrus, BA 41
5.42	11	44	−44	Left Cerebellar Tonsil
**LTA - LT**				
9.23	51	−19	−1	Left Inferior Frontal Gyrus, BA 47
6.61	64	29	6	Left Superior Temporal Gyrus, BA 42
4.12	44	−4	44	Left Middle Frontal Gyrus, BA 6
3.83	54	51	9	Left Middle Temporal Gyrus, BA 22
3.67	14	−46	41	Left Superior Frontal Gyrus, BA 8
3.25	−46	36	4	Right Superior Temporal Gyrus, BA 22
1.71	−36	56	24	Right Middle Temporal Gyrus, BA 39

*BA, Brodmann Area; S, straight driving; SA, straight driving with auditory distraction; L, left turns; R, right turns; LT, left turns with oncoming traffic; LTA, left turns with oncoming traffic and auditory distraction. Atlas coordinates L, left; P, posterior; I, inferior. Not all regions of significant activation are reported, with a cutoff value of *t* = 5 (unless there were less than 3 regions greater than this cutoff). Each peak within a given activation zone is only reported once.*

### Oculomotor Measures

**FIGURE 5:**
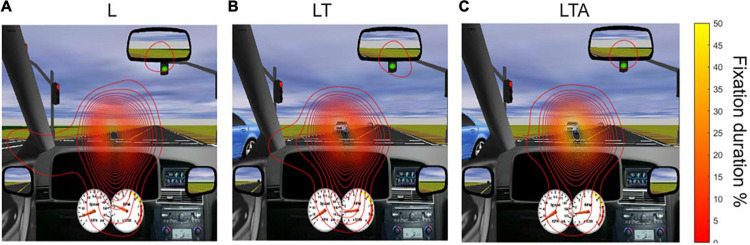
Distribution of gaze in the various tasks, averaged over all participants. Maps of percent fixation duration were computed as described in the text, and are shown as contour lines overlaid on the driving simulation screen. **(A)** L, left turn condition; **(B)** LT, left turn with traffic condition; and **(C)** LTA, left turns with traffic and auditory distraction.

Contour lines representing the spatial distribution of percent fixation duration for the L, LT and LTA conditions are shown in Figure 5, overlaid on the driving simulation environment. The distribution map for the L condition showed the widest span of fixation across the screen, including peripheral areas (mirror and speedometer) and the left side of the screen. The LT condition showed less fixation on the left side of the screen, as participants directed their gaze more to the oncoming traffic. The addition of auditory distraction in the LTA condition further narrowed the percent fixation duration onto the road immediately ahead.

Statistical analyses were subsequently conducted to test for the effects of driving condition on the spatial distribution of percent fixation duration. The sampling distributions of the peak amplitudes and widths of the percent fixation duration were normal for the L, LT, and LTA conditions (Kolmogorov-Smirnov Test, *p* < 0.05). No significant differences were found in the percent fixation duration distribution widths between these conditions, and no significant differences were found in the peak amplitudes of the fixation distribution between LT and L. However, the 95% CI testing of the mean difference of estimated peak amplitudes between “LTA – L” and “LTA – LT” rejected the null hypothesis. The peak amplitude for the LTA condition (56.8 (7.8)%) was found to be significantly greater than the values for both the LT condition (27.4 (4.5)%) and the L condition (25.4 (3.4)%) (95% CI Testing, Paired Samples *T*-Test, *p* < 0.05). The interpretation of this effect is that during the LTA condition, the spatial distribution of percent fixation duration for each participant was more consistent across the group than during the other two conditions.

**FIGURE 6:**
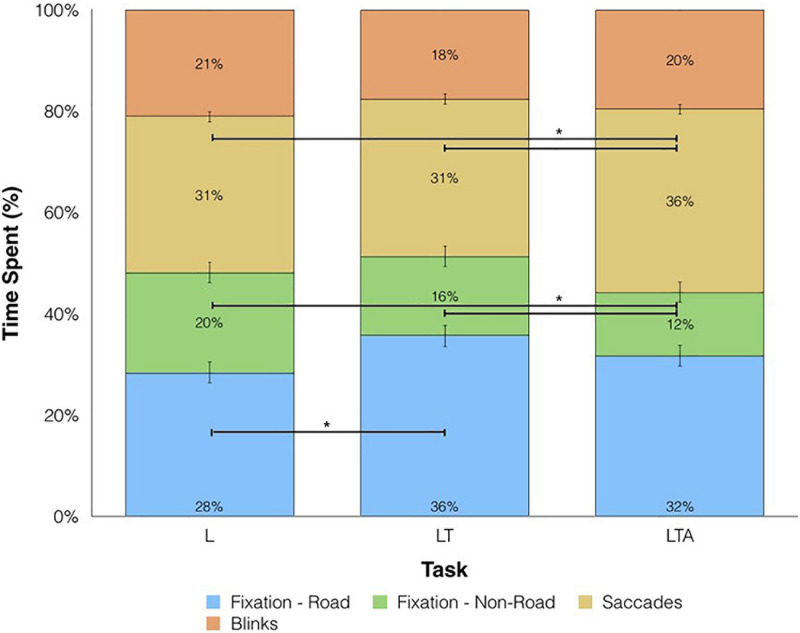
Percentage of time spent on eye movements for L, left turns; LT, left turns with oncoming traffic; and LTA, left turns with oncoming traffic and auditory distraction. Fixations on the road are shown in blue, fixations away from the road are shown in green, saccades are shown in yellow and blinks are shown in orange. Horizontal bars and asterisks represent statistically significant differences at *p* < 0.05.

Next, the average percentage of time spent performing each different eye behavior (fixations, saccades, blinks) was plotted in a stacked bar graph for each left turn task condition (Figure 6). No significant differences were found for the time spent fixating on the road (including motor vehicles) between LTA and LT conditions (Repeated Measures ANOVA; *p* > 0.05), whereas a significant elevation was found for LT compared to L (mean difference = 7.2%, Repeated Measures ANOVA; *p* < 0.05). Time spent fixating on non-road locations showed decreases with increasing task complexity, with mean differences of −7.3% and −3.3% for LTA compared to L and LTA compared to LT, respectively (Repeated Measures ANOVA; *p* < 0.05). The percentage of time performing saccades was significantly increased for LTA compared to LT and compared to L (mean difference = 5.4%, 5.0% respectively, Repeated Measures ANOVA; *p* < 0.05). No significant differences were observed between the peak velocity and number of saccades performed across the three tasks, however the mean saccade amplitude for the LTA condition (2.6 (0.28) units) was significantly less than that for the L condition (3.3 (0.35) units) (Repeated Measures ANOVA; *p* < 0.05).

In a separate analysis of eye-blink behavior (i.e., not reported in Figure 6), the mean blink rate over the total trial duration for the LTA condition [34.8 blinks/min (3.8)] was significantly lower than that for the L condition (37.0 blinks/min (5.3), Repeated Measures ANOVA; *p* < 0.05). No significant differences were found for the mean pupil dilation size for L, LT and LTA, although an increasing trend was observed across the conditions.

### Relationships Between Eye-Tracking Metrics and BOLD Signals

**FIGURE 7:**
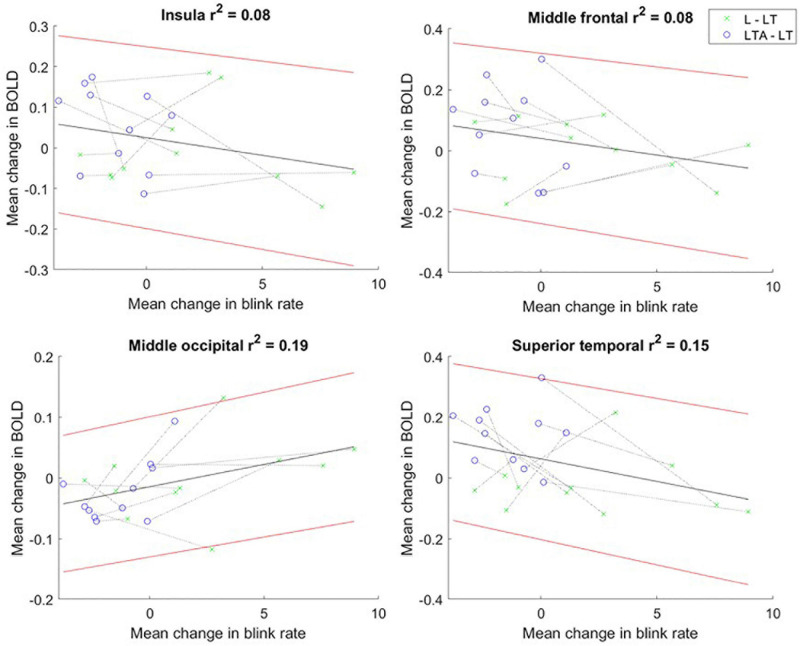
Mean change in BOLD fMRI signal vs. mean change in blink rate for “L – LT” (green) and “LTA – LT” (blue) task contrasts for the insula, and regions of interest in the middle frontal, middle occipital and superior temporal gyri. The dotted line connects the data points for each participant. The linear regression line is shown in black, with the 95% confidence interval for the regression line shown in red. BOLD, blood oxygenation level dependent; L, left turns; LT, left turns with oncoming traffic; LTA, left turns with oncoming traffic and auditory distraction.

No significant relationships were found between the mean BOLD signal intensity and pupil dilation size. However, scatter plots showing the change scores for the mean BOLD signal intensity and blink rate (L – LT, LTA – LT) showed negative, statistically significant Pearson correlations in the insula [mean slope of −0.13 (0.05)], middle frontal [−0.01 (0.04)] and superior temporal [−0.03 (0.07)] regions, as shown in Figure 7. A positive, statistically significant Pearson correlation was found in the middle occipital gyrus [0.02 (0.02)).

## Discussion

The present study investigated simulated driving using the novel combination of fMRI and eye-tracking. As the study was intended to investigate whether it was possible to replicate the fMRI findings of Schweizer et al. (2013) using the same task design, comparisons to this prior work are made throughout. In addition, given that driving requires considerable visual processing demands, the study extends beyond Schweizer et al. (2013) to include eye-tracking for an initial evaluation of oculomotor measures for each task, and their relationship with simulated driving task-related behavior and brain activity. The results of the study are in good agreement with the stated hypotheses, and are discussed below for the behavioral metrics of the driving simulator performance, brain activity and oculomotor measures. The inter-relationships between these measures are then discussed, followed by the limitations of the study.

### Behavioral Metrics of Simulated Driving

The behavioral metrics of simulated driving performance (Table 1) indicated that the R, L, LT, and LTA conditions were performed well by participants for the most part, while they obeyed the “rules of the road.” Over all participants and fMRI runs, only two collisions were observed for both the LT and LTA conditions, whereas only one collision was observed for the L condition. Only one participant was involved in multiple collisions (one in each of the R, LT, and LTA conditions). The left turn conditions were performed similarly by all participants on the time to turn metric, and the turn speed was similar in the L and LT conditions. In addition, the S and SA conditions were performed without differences in lane position. These results suggest that each of the simulated driving conditions, while containing variations in the complexity of task, were well within the skill level for efficient execution by the participants without large changes in the behavioral metrics studied. It is important to emphasize that these results are specific to young healthy drivers, and analogous results for other populations of interest, such as elderly drivers or individuals with cognitive impairments, are likely to be different.

Two noteworthy differences in simulated driving performance were found between certain conditions. First, the turn speed was significantly slower for the R condition than for the L, LT and LTA conditions. This may have resulted from the smaller turning radius of the road when turning right, resulting in slower turn speeds to drive safely and maintain lane position. However, the R and L conditions showed similar brain activation maps (see below), suggesting that both turns require similar behavioral elements. Second, there were slight but significant reductions in driving speed associated with distracted driving. The effect was slightly more pronounced when comparing the SA and S conditions, whereas the slowing in the LTA condition was only significant in comparison to the L condition, not the LT condition. These results suggest that the primary task (driving) was slightly impacted by the additional cognitive demand associated with responding to the secondary task (auditory questions) in both cases. From the auditory task performance, there was no evidence that participants were making trade-offs between how well to perform the driving tasks in comparison to the auditory task – as the question response time and response accuracy was not statistically significant between the LTA and SA conditions. This may be due to the age of the participants, or the auditory tasks that were administered (general knowledge true or false questions), as they were not very demanding. This effect on driving speed could be investigated in more detail in the future by conducting a behavioral study with more participants, and by manipulating the strength of the auditory distraction. Certain behavioral data related to the LTA condition were also noteworthy: the question response time was substantially shorter than the time to turn, and the time to turn was not significantly different from that of the LT condition. As described in the Results, these findings suggest that participants engaged in driving-auditory dual-tasking in the LTA condition rather than performing the two tasks sequentially.

The results observed in this study were similar to those previously reported by Schweizer et al. (2013), however some slight discrepancies were also observed. In both studies, no significant differences were found between the lane position in straight driving and distracted straight driving. The average response accuracy to the audio distraction task was also similar, and the observed turn speed of right turns was slower than all left turns (L, LT and LTA). In this study, the turn speed of the L condition was faster than both LT and LTA, whereas Schweizer et al. (2013) reported the turn speed of the L condition was slower than both LT and LTA. Additionally, no significant differences were previously observed in the average speed during straight driving and distracted straight driving. It is reasonable to speculate that these discrepancies were observed due to the different cohorts investigated in the two studies, the relatively low sample size and the variability in human behavior. None of the participants volunteered in both studies. Although the two studies were conducted using slightly different versions of fMRI-compatible driving simulator, one-hour simulator training periods were part of the experimental design in both cases to control for systematic bias. Irrespective of the discrepancies, the simulated driving behavior in the present study helps to inform the interpretation of the task-related brain activation maps, as discussed below.

### Brain Activity

Previous simulated driving studies have shown a network of brain regions activated during straight driving, including the occipital and parietal lobes, cerebellum, and areas involved in motor control and somatosensory integration (Calhoun et al., 2002; Uchiyama et al., 2003; Graydon et al., 2004; Spiers and Maguire, 2007; Just et al., 2008; Callan et al., 2009; Crundall and Underwood, 2011; Calhoun and Pearlson, 2012; Li et al., 2012; Schweizer et al., 2013; Hyung-Sik et al., 2014; Choi et al., 2017; Ware et al., 2020). Straight driving involves visual vigilance and small adjustments in driving control to maintain speed and lane position. To isolate the visual, motor and cognitive functions activated in more demanding driving tasks (e.g., performing turns), straight driving was chosen as the baseline condition. As such, all the subsequent activation maps of the task conditions must be thought of as referenced to this baseline behavior, and represent an augmentation of this supposedly least-taxing state of driving and associated brain activity.

In this study, occipital, parietal, cerebellar and motor areas were recruited in both the R and L conditions, and also the LT condition (Figure 2). The brain regions that were found to be activated (occipital, parietal, cerebellar and motor areas) were consistent with previous studies investigating simple driving as noted above (Calhoun et al., 2002; Uchiyama et al., 2003; Graydon et al., 2004; Spiers and Maguire, 2007; Callan et al., 2009; Crundall and Underwood, 2011; Calhoun and Pearlson, 2012; Li et al., 2012; Hyung-Sik et al., 2014; Choi et al., 2017; Ware et al., 2020). Some additional aspects are noteworthy. First, the driving conditions showed decreased activity in the medial frontal cortex and anterior cingulate cortex (ACC). Both regions are part of the default mode network (DMN), which decreases in activity when performing tasks (Raichle, 2015). Thus, it is speculated that the more engaging R, L, and LT conditions produced more suppression of the DMN than occurred during straight driving. Second, minimal activity was observed in the rest of the prefrontal cortex, suggesting that the R, L, and LT conditions were performed by the participants as a consequence of extensive training and practice in driving maneuvers, leading to efficient mental processing controlled primarily by the posterior brain, largely in the absence of executive control. Third, no significant differences were found in the brain activation between R, L and LT conditions. The lack of difference in brain activation between R and L conditions is expected because the task requirements are largely the same, except that the direction of simulated movement is different. Additionally, the lack of difference in brain activation between the LT and L or R conditions is consistent with the behavioral metrics, which showed that each condition produced a similar time of turning. This suggests that the inclusion of traffic to left turns was not very challenging for this cohort of young but experienced drivers.

The present results are similar to but slightly different from those reported by Schweizer et al. (2013), in which the R condition produced minimal significant activations, including the somatosensory association, parietal and visual cortices, whereas the L condition showed greater activation in the posterior regions (occipital, parietal, motor, cerebellar), and the LT condition showed a progressive increase in the extent of activation in the posterior network and cingulate cortex. Two methodological elements are noteworthy in discussing the findings of this prior study in relation to the present work. First, the differences in brain activation between conditions were assessed visually and qualitatively in the prior study, whereas the present study used a quantitative assessment of statistical contrasts. Thus, it is possible that effects interpreted between conditions in the previous study were not statistically significant. Second, as previously discussed in section 4.1 above, the two studies consist of different samples of young healthy adults. Although the two studies were conducted using slightly different versions of 3T Siemens MRI system, systematic differences arising from this effect are anticipated to be minor when comparing task-related brain activity at the group level.

Shifting to the effects of distraction on brain activity, brain activations were observed in the middle and superior temporal gyrus (MTG, STG) when the SA – S contrast was evaluated (Figure 3A). These regions have previously been associated with cognitive processes including language and semantic processing, spatial awareness, and attentional processing from the dorsal and ventral streams (Chao et al., 1999; Tranel et al., 1997; Onitsuka et al., 2004). Similar effects were observed for the LTA – S contrast (Figure 3B). Furthermore, the LTA – SA contrast showed activation of the STG, occipital, primary motor and premotor cortices, whereas significant decreases in activations were observed in the left inferior frontal gyrus (IFG) (Figure 4A). The activations in the occipital, primary motor and premotor cortices are consistent with the increased visuomotor demands associated with performing left turns with oncoming traffic in the LTA condition in relation to the straight driving required in the SA condition. Greater activation in the STG suggests a higher demand for cognitive processing in the LTA condition in relation to the SA condition. In addition, the LTA condition showed less activation of the left IFG compared to the SA condition. The left IFG has been linked to language processing and working memory, and has been found to be activated during semantic tasks (Petersen et al., 1989; Démonet et al., 1992; Moss et al., 2005; Liakakis et al., 2011). Two possible mechanisms may be speculated for the observed decrease in activation, although future work with a larger sample size will be required to determine which is correct. From Table 1, there was a trend for lower response accuracy for the auditory task in the LTA condition compared to the SA condition, but without statistical significance. Considering this trend as a “false positive,” then the difference in IFG activity (and inferred resources for language processing) should disappear with larger sample size. Alternatively, if the trend is thought of as a “false negative,” then both the difference in response accuracy and IFG activity should persist in a study with larger sample size. As the task instructions were not specific about prioritizing the auditory task, it is conceivable that participants performed as they would in the real world, to trade off auditory task performance to ensure safe driving behavior in the more cognitively demanding task (LTA compared to SA). The observed trend in response accuracy is consistent with this interpretation, although no more definite statements can be made at present.

Distraction poses a great risk to drivers especially when they perform left turns. Of the MVCs that occur while drivers turn or cross an intersection, 61% involve turning in the left direction (2008). Compared to L or LT conditions, the LTA condition showed significantly greater bilateral activation in the middle frontal gyrus (MFG), IFG, SFG, MTG, STG and ACC (Figure 4B). In particular, the increase in frontal activity is characteristic of dual-tasking (Dux et al., 2006) and is consistent with the hypotheses of the study. The right MFG and IFG have been associated with the ventral attention network, activated in response to unexpected stimuli and to attend to relevant task information and distractors (Friedman-Hill et al., 2003; Hahn et al., 2006; Buschman and Miller, 2007; Fan et al., 2008; Neufang et al., 2011; Weissman and Prado, 2012; Japee et al., 2015). The IFG has also been suggested to be play a role in sustained attention, attentional control, working memory and motor response inhibition (Swick et al., 2008; Hampshire et al., 2010; Tops and Boksem, 2011). The activation of this region suggests the involvement of attentional control and reorientation when a secondary task was introduced. The left SFG is known to play a role in working memory and executive processing, suggesting that recruitment of this region is necessary for performance of the auditory task (Boisgueheneuc et al., 2006). Recruitment of the MTG and STG suggests involvement of spatial awareness and attentional processing to undertake executive functions and additional task processing, consistent with previous findings (Schweizer et al., 2013). The ACC has been linked to cognitive modulation and error processing (Bush et al., 2000; Paus, 2001; Fan et al., 2008; Orr and Hester, 2012). Greater activation in the ACC suggests that the secondary auditory task increased the overall cognitive load, and thus the need to monitor and correct for errors.

In addition to the regions of increased activation in the LTA condition compared to the LT or L conditions, significantly decreased activation was observed in the right middle occipital gyrus (MOG), superior occipital gyrus (SOG), right inferior parietal lobe (IPL), cerebellum and precuneus. This observation is also in line with the study hypotheses. The right MOG and SOG are considered to be part of the visual dorsal stream, associated with spatial and visuomotor processing (Dumoulin et al., 2000; Wandell et al., 2007; Matthys et al., 2009; Renier et al., 2010), and the right IPL is involved in sustained visual and somatosensory attention (Wilkins et al., 1987; Pardo et al., 1991; Rueckert and Grafman, 1996, 1998). These results are in agreement with those of previous studies that investigated brain activity in simulated driving with a relatively simple secondary behavioral task, showing a shift in activation from the posterior, visual areas to the frontal areas responsible for planning, decision-making and cognition (Just et al., 2008; Schweizer et al., 2013; Choi et al., 2017).

### Oculomotor Measures

Investigating the same driving tasks as in the present work, Schweizer et al. (2013) stated that “the distracted brain sacrificed areas in the posterior brain important for visual attention and alertness to recruit enough brain resources to perform a secondary cognitive task.” However, the inclusion of oculomotor measures in the present study, as subsequently described below, suggest an additional, alternative interpretation. Given the task instructions, participants modified their gaze behavior to maintain the ability to make left turns in the presence of oncoming traffic. As the task was performed in a simulator under very simple conditions, there were no significant task penalties associated with not gazing at off-road areas such as the dash-board and rear-view mirror. Instead, in the presence of distraction there were performance advantages to directing gaze much more consistently at the oncoming traffic to simplify the visual processing demands (and the resulting brain activity). Additional experimental methodology involving augmented simulated driving tasks will be required to evaluate these two interpretations further.

There is a correlation between distraction and increased risk of crashes and near-crashes (2006). The implication of interest here is that one of the effects of distraction, contributing to its role in dangerous driving, is an associated modification in visual processing that can potentially stop drivers from reacting to possible threats. Supporting the modulation of visual processing in the present study, and as hypothesized, it was found that the less demanding left turn task (i.e., L condition) resulted in a wide distribution of gaze points across the scene as quantified using the percent fixation duration map, including peripheral areas (mirror and speedometer) and the left side of the road (Figure 5); as the task became more demanding, (i.e., LT condition), the distribution remained similar, although there was an increase in the fraction of time spent fixating on the road at oncoming traffic, as appropriate for the specific task (Figure 6). With the introduction of distraction (i.e., the LTA condition), the fixation distribution narrowed: the fixation duration onto the immediate road and oncoming vehicles was increased further, as participants concentrated on the essential features of the visual scene to maintain driving performance. Although differences in the widths of the fixation distribution were not significantly different (LTA – LT, LTA – L), the effect was nevertheless detected as an increase in the mean peak amplitude of the fixation distribution for these contrasts. This suggests that as a group, the participants were gazing more consistently at the oncoming traffic during the LTA condition. Although not specifically studied in these driving tasks, it would be useful in future studies to investigate whether these changes in gaze behavior result in decreased ability to react appropriately to other dynamic changes in the visual scene that would constitute a threat to safe driving - such as cross-traffic failing to obey traffic lights – requiring aversive maneuvers. Furthermore, the current methodology and study findings are not definitive regarding how to quantify attention (and modulations thereof) across the driving task conditions, and this should be addressed in future studies.

Irrespective of the two interpretations discussed above, the oculomotor results are consistent with the brain activation maps showing a decrease in activation in the visual cortex for the LTA – LT contrast (Figure 4B). Figures 5, 6 confirm that this reduction is accompanied by a narrowed field of view, involving an increasing trend in the percentage of on-road fixations and a significant decrease in the percentage of off-road fixations when comparing LTA to LT. Furthermore, compared to the LT and L conditions, the LTA condition showed less percentage time of overall fixations (i.e., road and non-road) with significantly greater percentage of time spent performing saccades. This observation is consistent with recent eye-tracking research showing that the percentage of time spent in fixation can decrease when visual processing tasks are performed in the presence of auditory distraction tasks (Wu et al., 2019). Control of saccades has been associated with the frontal eye fields (Brodmann Area 8), the dorsolateral prefrontal cortex (DLPFC) and the cingulate eye field within the ACC (Künzle et al., 1976; Vernet et al., 2014). In Figure 4B, increased activation was observed in these regions when comparing the LTA condition to the LT condition, consistent with increased saccades in the former. Together with the decrease in fixation on the periphery in the LTA condition, these results further support the interpretations that for the simulated driving tasks investigated, distraction resulted in less visual processing resources (a) made available or (b) required to support driving performance.

No significant differences were found in blink rate for the LT – L contrast. This is consistent with the brain activation maps and behavioral results, which did not show significant differences between the two tasks. As hypothesized, however, distraction resulted in a statistically significant decrease in blink rate for the LTA – L contrast. This finding is consistent with consensus in the literature that blink rate decreases with increased cognitive load (Brookings et al., 1996; Ohira, 1996; Van Orden et al., 2001; Haak et al., 2009; McIntire et al., 2014; Faure et al., 2016; Maffei and Angrilli, 2018). However, one study found an increase in blink rate when a straight driving task was combined with an auditory arithmetic task - suggesting that other factors may influence the effect, such as disinhibition of blink rate effects under certain task conditions and ranges of workload (Tsai et al., 2007). The experimental design of the present study cannot refute this claim.

A trend of increasing pupil diameter was observed across the left turn conditions (L, LT, LTA) suggesting that greater cognitive processing may have occurred across this progression of tasks. Although this effect was small and not statistically significant, the direction of the effect is consistent with the brain activation data, and with the study hypotheses. Pupil dilation in cognitive processing has been linked to activation of the MFG and ACC, measuring attention and effort (Siegle et al., 2003; Alnaes et al., 2014; Murphy et al., 2014; Zekveld et al., 2014; Joshi et al., 2016). This is consistent with the increase in activation of the MFG and ACC observed in the brain activation maps for the LTA - LT contrast (Figure 4B).

### Relationships Between Eye-Tracking Measures and Bold Signals

The change score relationships for BOLD signals and eye-tracking metrics were invested as r^2^ values, representing the proportion of variance of the change in BOLD signal that can be attributed to the change in each eye-tracking metric. As hypothesized, significant Pearson correlations were found between the BOLD signal change score and blink rate change score for several regions. Negative correlations were found for the insula, the STG and the MFG, whereas a positive correlation was found for the MOG. The insula and STG have been described to play a role in the suppression of eye blinking (Lerner et al., 2009; Berman et al., 2012). The MFG, which has been associated with the reorientation of attention, has also been correlated with blink inhibition (Andersson et al., 2009; Mazzone et al., 2010; Neufang et al., 2011; Japee et al., 2015). The increased activation in the insula, STG and MFG is consistent with the suppression of the blink mechanism as the cognitive load is increased. The MOG has been related to visual attention, spatial and visuomotor processing, suggesting a decrease in one, two, or all of these components across the driving tasks in step with the associated decrease in blink rate (Mangun et al., 1998; LaBerge, 2000; Hahn et al., 2006; Wandell et al., 2007; Renier et al., 2010). However, it must be noted that the observed r^2^ values were modest for all four brain regions. In addition to the natural variations in human performance and brain function, the BOLD signal is influenced by numerous confounding factors, including age, sex and caffeine levels (D’Esposito et al., 2003; Chang et al., 2018). This can result in high inter-subject variability, reducing the power of the fMRI data. More work will be required to determine the main factor underlying the change in BOLD signal related to oculomotor behavior in the present context, involving a larger sample size and/or different analysis tools. Despite this, the observed relationships are important to note nevertheless. They support the interpretation that an increase in cognitive load across L, LT and LTA tasks increases certain brain activity to suppress blink rate; and also results in decreasing activity related to aspects of visual processing – suggesting a strategy of using neural resources efficiently to maintain task performance.

### Limitations

The results of the present study must be considered in relation to a number of limitations in the experimental design and execution. Technical issues associated with fMRI-compatible eye-tracking caused the sample size to be somewhat lower than originally intended. One disadvantage of eye-tracking is that participant factors (e.g., glasses, contacts, eye color) can negatively impact how the pupil or the eye are tracked from the video recordings (Jacob and Karn, 2003; Nyström et al., 2013; Kok and Jarodzka, 2017). Additionally, cognitive processes cannot be inferred directly and conclusively from eye-movements. Visual attention can also be guided by peripheral vision, which cannot be captured through eye-tracking (Kok and Jarodzka, 2017). Just as it is possible to attend to something without directly fixating on it, it is also possible to fixate on something without attending to it - an effect known as attentional blindness (Mack, 2003). As a result, it can be challenging to develop a complete understanding of the relationships between eye movements and cognition. Thus, the eye-tracking results in the present study must be viewed as supporting (rather than defining) the interpretation of brain/behavior relationships across the various driving conditions.

Although the results largely replicate (while also extending from) a previous fMRI study with a very similar experimental design (Schweizer et al., 2013), additional work involving a larger sample size would be beneficial to better characterize human variability and potentially to detect important new findings with potentially smaller effect sizes in the driving conditions studied. The current study also examines the effect of distraction in a young adult population, which is like to differ when conducting analogous work involving the elderly. When distracted, older drivers have been found to drive slower with increased steering variability, and to commit more driving errors (Thompson et al., 2012). Although MVCs more frequently involve younger drivers (possibly due to lack of sufficient driving skills and incomplete maturation), older drivers have been attributed to an increased risk of MVCs in highly complex situations (e.g., intersections) due to age-associated changes in attention and cognitive decline (Stinchcombe and Gagnon, 2013). In addition, deficits in visual functioning, perception and information processing have been observed in the elderly (Kline et al., 1992; Stinchcombe and Gagnon, 2013). However, the present study of young adults is useful, as distracted driving is a prominent issue in this demographic with the rise in cell phone technology and social media (Klauer et al., 2014). This study also provides a normative data set to be used as a baseline for future studies.

The eye-tracking data obtained in this study were useful, but did not exhibit sufficient detection power to reveal statistically significant effects in all cases. In this study cohort, an increasing trend was seen in the pupil dilation size with the increase in cognitive load, although the effect was not significant. If this work were to be extended to an older adult or clinical population with cognitive deficits, this eye-tracking metric will remain important to investigate, as eye-tracking has been suggested for assessment of cognitive impairment and diagnosis of neurodegenerative conditions. Patients with dementia and Alzheimer’s disease have been shown to demonstrate altered pupillary responses and oculomotor impairments, which can be associated to brain function and neural mechanisms to assess cognitive dysfunction (Pavisic et al., 2017; Marandi and Gazerani, 2019; Tao et al., 2020). Aging has also been associated with an increased latency of saccades and increased blink frequency in addition to neurocognitive decline (Marandi and Gazerani, 2019). Thus, the inclusion of eye-tracking methodology to future simulated driving studies may provide critical insight to the understanding of brain activity and behavior.

Another limitation of this study is that the simulation used did not replicate potential anxiety experienced in real driving due to the lack of stressing factors (e.g., other drivers, no real crash risk or danger). A subsequent experiment may expand on this area by incorporating more factors into the simulation such as vehicles to maintain a safe following distance, multiple lanes on the road with other vehicles, increased traffic or construction; as well as narratives, rewards and penalties related to driving performance.

Lastly, the simulated driving apparatus used inside the MRI system inevitably produced an experience that was different from real world driving, as the participants viewed the simulation through a projector screen while lying on a patient table, driving with minimal head and body motion. Usage of driving simulator hardware is suggested to provide a sufficient level of realism for evaluating demanding driving scenarios that would be dangerous to assess in on-road testing (Kan et al., 2012). However, the setup may create activation maps for simulated driving that imperfectly reflect the mental processes that occur when individuals drive motor vehicles in the real world (Kan et al., 2012). To mitigate this possibility, each participant underwent a training session with the driving simulator prior to fMRI. The training session was intended to familiarize participants with the simulator controls and thus ensure that their driving skills encompassed use of the experimental apparatus.

## Conclusion

The present study uses a unique multi-measurement approach, combining fMRI with eye-tracking to measure brain and oculomotor behavior during simulated driving. It was observed that simple driving primarily involved the visual and motor systems, whereas the introduction of auditory distraction shifted resources to the frontal and temporal systems for greater cognitive processing. Neural resources were modulated as task complexity increased, with re-allocation of resources when there were competing task demands in driving with auditory distraction. The inclusion of eye-tracking data furthered understanding of the effect of distraction on driving behavior and brain activity. Decreased brain activation in the visual system for distracted driving was supported by a more peaked mean distribution of gaze points as quantified by the percent fixation duration metric, concentrated on the road at the expense of the periphery, as well as a decrease in blink rate. Providing a baseline for comparison, these findings may be applied to future research involving an elderly or clinical population to determine the effect of aging or brain damage on the ability to multitask while driving. Because distraction promotes dangerous driving behavior and has been shown to display significant changes in brain activation and eye movements, assessing fitness to drive should consider including varying driving demands (e.g., with a secondary task introduced as distraction) to mimic real world scenarios where distraction is prevalent. Automotive companies may also wish to consider implementing strategies to mitigate visual tunneling when distracted, or to minimize the effect of distracting activities and communication devices.

## Data Availability Statement

The raw data supporting the conclusions of this article will be made available by the authors, without undue reservation.

## Ethics Statement

The studies involving human participants were reviewed and approved by the Research Ethics Board at Sunnybrook Health Sciences Centre, Canada. The patients/participants provided their written informed consent to participate in this study.

## Author Contributions

TS and SG did the conceptualization and supervision. NY, FT, and SG did the investigation. SG did the resources, project administration, and funding acquisition. NY and SG did the data curation and writing-original draft preparation. NY, FT, NC, TS, and SG wrote-reviewed and edited and did the visualization. All authors contributed to the article and approved the submitted version.

## Conflict of Interest

The authors declare that the research was conducted in the absence of any commercial or financial relationships that could be construed as a potential conflict of interest.

## Publisher’s Note

All claims expressed in this article are solely those of the authors and do not necessarily represent those of their affiliated organizations, or those of the publisher, the editors and the reviewers. Any product that may be evaluated in this article, or claim that may be made by its manufacturer, is not guaranteed or endorsed by the publisher.

## References

[B1] Alnaes D. Sneve M. H. Espeseth T. Endestad T. van de Pavert S. H. P. Laeng B. (2014). Pupil size signals mental effort deployed during multiple object tracking and predicts brain activity in the dorsal attention network and the locus coeruleus. *J. Vis.* 14:1. 10.1167/14.4.1 24692319

[B2] Andersson M. Ystad M. Lundervold A. Lundervold A. J. (2009). Correlations between measures of executive attention and cortical thickness of left posterior middle frontal gyrus - a dichotic listening study. *Behav. Brain Funct.* 5:41. 10.1186/1744-9081-5-41 19796388 PMC2761925

[B3] Berman B. D. Horovitz S. G. Morel B. Hallett M. (2012). Neural correlates of blink suppression and the buildup of a natural bodily urge. *Neuroimage* 59 1441–1450. 10.1016/j.neuroimage.2011.08.050 21906689 PMC3230735

[B4] Boisgueheneuc F. D. Levy R. Volle E. Seassau M. Duffau H. Kinkingnehun S. (2006). Functions of the left superior frontal gyrus in humans: a lesion study. *Brain* 129 3315–3328. 10.1093/brain/awl244 16984899

[B5] Bowers A. R. Anastasio R. J. Sheldon B. S. O’Connor M. G. Hollis A. M. Howe P. D. (2013). Can we improve clinical prediction of at-risk older drivers? *Accident Anal. Prevent.* 59 537–547. 10.1016/j.aap.2013.06.037 23954688 PMC3769510

[B6] Brookings J. B. Wilson G. F. Swain C. R. (1996). Psychophysiological responses to changes in workload during simulated air traffic control. *Biol. Psychol.* 42 361–377. 10.1016/0301-0511(95)05167-8 8652753

[B7] Buschman T. J. Miller E. K. (2007). Top-Down versus bottom-up control of attention in the prefrontal and posterior parietal cortices. *Science* 315 1860–1862. 10.1126/science.1138071 17395832

[B8] Bush G. Luu P. Posner M. I. (2000). Cognitive and emotional influences in anterior cingulate cortex. *Trends Cogn. Sci.* 4 215–222. 10.1016/s1364-6613(00)01483-2 10827444

[B9] Calhoun V. D. Pearlson G. D. (2012). A selective review of simulated driving studies: combining naturalistic and hybrid paradigms, analysis approaches, and future directions. *NeuroImage* 59 25–35. 10.1016/j.neuroimage.2011.06.037 21718791 PMC3197797

[B10] Calhoun V. D. Pekar J. J. McGinty V. B. Adali T. Watson T. D. Pearlson G. D. (2002). Different activation dynamics in multiple neural systems during simulated driving. *Hum. Brain Mapp.* 16 158–167. 10.1002/hbm.10032 12112769 PMC6872105

[B11] Callan A. M. Osu R. Yamagishi Y. Callan D. E. Inoue N. (2009). Neural correlates of resolving uncertainty in driver’s decision making. *Hum. Brain Mapp.* 30 2804–2812. 10.1002/hbm.20710 19117274 PMC6871010

[B12] Cantin V. Lavallière M. Simoneau M. Teasdale N. (2009). Mental workload when driving in a simulator: effects of age and driving complexity. *Accident Anal. Prevent.* 41 763–771. 10.1016/j.aap.2009.03.019 19540965

[B13] Chang D. Song D. Zhang J. Shang Y. Ge Q. Wang Z. (2018). Caffeine caused a widespread increase of resting brain entropy. *Sci. Rep.* 8:2700. 10.1038/s41598-018-21008-6 PMC5807546 29426918

[B14] Chao L. L. Haxby J. V. Martin A. (1999). Attribute-based neural substrates in temporal cortex for perceiving and knowing about objects. *Nat. Neurosci.* 2 913–919. 10.1038/13217 10491613

[B15] Choi M.-H. Kim H.-S. Yoon H.-J. Lee J.-C. Baek J.-H. Choi J.-S. (2017). Increase in brain activation due to sub-tasks during driving: fMRI study using new MR-compatible driving simulator. *J. Physiol. Anthropol.* 36:11. 10.1186/s40101-017-0128-8 PMC5270359 28126038

[B16] Cox R. (1996). AFNI: software for analysis and visualization of functional magnetic resonance neuroimages. *Comp. Biomed. Res.* 29 162–173. 10.1006/cbmr.1996.0014 8812068

[B17] Crundall D. Underwood G. (2011). “Visual attention while driving,” in *Handbook of Traffic Psychology*, ed. Porter B. E. (Amsterdam: Elsevier), 137–148. 10.1016/b978-0-12-381984-0.10011-6

[B18] Démonet J. F. Chollet F. Ramsay S. Cardebat D. Nespoulous J. L. Wise R. (1992). The anatomy of phonological and semantic processing in normal subjects. *Brain* 115 (Pt 6) 1753–1768. 10.1093/brain/115.6.1753 1486459

[B19] D’Esposito M. Deouell L. Y. Gazzaley A. (2003). Alterations in the BOLD fMRI signal with ageing and disease: a challenge for neuroimaging. *Nat. Rev. Neurosci.* 4 863–872. 10.1038/nrn1246 14595398

[B20] Drews F. A. Yazdani H. Godfrey C. N. Cooper J. M. Strayer D. L. (2009). Text messaging during simulated driving. *Hum. Factors* 51 762–770. 10.1177/0018720809353319 20196300

[B21] Dumoulin S. O. Bittar R. G. Kabani N. J. Baker C. L. Le Goualher G. Bruce Pike G. (2000). A new anatomical landmark for reliable identification of human area V5/MT: a quantitative analysis of sulcal patterning. *Cereb. Cortex* 10 454–463. 10.1093/cercor/10.5.454 10847595

[B22] Dux P. E. Ivanoff J. Asplund C. L. Marois R. (2006). isolation of a central bottleneck of information processing with time-resolved fMRI. *Neuron* 52 1109–1120. 10.1016/j.neuron.2006.11.009 17178412 PMC2527865

[B23] Fan J. Hof P. R. Guise K. G. Fossella J. A. Posner M. I. (2008). The functional integration of the anterior cingulate cortex during conflict processing. *Cereb. Cortex* 18 796–805. 10.1093/cercor/bhm125 17652463

[B24] Faure V. Lobjois R. Benguigui N. (2016). The effects of driving environment complexity and dual tasking on drivers’ mental workload and eye blink behavior. *Transportation Res. Part F: Traffic Psychol. Behav.* 40 78–90. 10.1016/j.trf.2016.04.007

[B25] Fitch G. M. Bartholomew P. R. Hanowski R. J. Perez M. A. (2015). Drivers’ visual behaviour when using handheld and hands-free cell phones. *J. Safety Res.* 54 105–108.e29. 10.1016/j.jsr.2015.06.008 26403895

[B26] Fraade-Blanar L. A. Ebel B. E. Larson E. B. Sears J. M. Thompson H. J. Chan K. C. G. (2018). Cognitive decline and older driver crash risk. *J. Am. Geriatr. Soc.* 66 1075–1081. 10.1111/jgs.15378 29667168 PMC6541224

[B27] Friedman-Hill S. R. Robertson L. C. Desimone R. Ungerleider L. G. (2003). Posterior parietal cortex and the filtering of distractors. *Proc. Natl. Acad. Sci. U S A.* 100 4263–4268. 10.1073/pnas.0730772100 12646699 PMC153081

[B28] Gable T. M. Kun A. L. Walker B. N. Winton R. J. (2015). “Comparing heart rate and pupil size as objective measures of workload in the driving context: initial look,” in *Proceedings of the Adjunct Proceedings of the 7th International Conference on Automotive User Interfaces and Interactive Vehicular Applications - Automotive UI’15*, (Nottingham: ACM Press), 20–25.

[B29] Genovese C. R. Lazar N. A. Nichols T. (2002). Thresholding of statistical maps in functional neuroimaging using the false discovery rate. *NeuroImage* 15 870–878. 10.1006/nimg.2001.1037 11906227

[B30] Graydon F. X. Young R. Benton M. D. Genik R. J. Posse S. Hsieh L. (2004). Visual event detection during simulated driving: identifying the neural correlates with functional neuroimaging. *Transportation Res. Part F: Traffic Psychol. Behav.* 7 271–286. 10.1016/j.trf.2004.09.006

[B31] Haak M. Bos S. Panic S. Rothkrantz L. J. M. (2009). “Detecting stress using eye blinks and brain activity from EEG signals,” in *Proceeding of the 1st Driver Car Interaction and Interface (DCII 2008)*, Netherland 35–60.

[B32] Hahn B. Ross T. J. Stein E. A. (2006). Neuroanatomical dissociation between bottom–up and top–down processes of visuospatial selective attention. *NeuroImage* 32 842–853. 10.1016/j.neuroimage.2006.04.177 16757180 PMC2652125

[B33] Hamish Jamson A. Merat N. (2005). Surrogate in-vehicle information systems and driver behaviour: effects of visual and cognitive load in simulated rural driving. *Transportation Res. Part F: Traffic Psychol. Behav.* 8 79–96. 10.1016/j.trf.2005.04.002

[B34] Hampshire A. Chamberlain S. R. Monti M. M. Duncan J. Owen A. M. (2010). The role of the right inferior frontal gyrus: inhibition and attentional control. *Neuroimage* 50 1313–1319. 10.1016/j.neuroimage.2009.12.109 20056157 PMC2845804

[B35] Hird M. A. Vesely K. A. Fischer C. E. Graham S. J. Naglie G. Schweizer T. A. (2017). Investigating simulated driving errors in amnestic single- and multiple-domain mild cognitive impairment. *J. Alzheimer’s Dis.* 56 447–452. 10.3233/jad-160995 27983557

[B36] Holland M. K. Tarlow G. (1972). Blinking and mental load. *Phych. Rep*. 31 119–127. 10.2466/pr0.1972.31.1.119 5055889

[B37] Hyung-Sik K. Mi-Hyun C. Hee-Jeong Y. Hyun-Joo K. Ul-Ho J. Sung-Jun P. (2014). Cerebral activation and lateralization due to the cognition of a various driving speed difference: an fMRI study. *Biomed. Mater. Eng.* 24 1133–1139. 10.3233/bme-130913 24212006

[B38] Jacob R. J. K. Karn K. S. (2003). “Eye tracking in human-computer interaction and usability research: ready to deliver the promises,” in *The Mind’s Eye: Cognitive and Applied Aspects of Eye Movement Research*, eds Deubel H. Hyönä J. R. I. (Amsterdam: Elsevier Science).

[B39] Japee S. Holiday K. Satyshur M. D. Mukai I. Ungerleider L. G. (2015). A role of right middle frontal gyrus in reorienting of attention: a case study. *Front. Syst. Neurosci.* 9:23. 10.3389/fnsys.2015.00023 25784862 PMC4347607

[B40] Joshi S. Li Y. Kalwani R. M. Gold J. I. (2016). Relationships between pupil diameter and neuronal activity in the locus coeruleus. colliculi, and cingulate cortex. *Neuron* 89 221–234. 10.1016/j.neuron.2015.11.028 26711118 PMC4707070

[B41] Just M. A. Keller T. A. Cynkar J. (2008). A decrease in brain activation associated with driving when listening to someone speak. *Brain Res.* 1205 70–80. 10.1016/j.brainres.2007.12.075 18353285 PMC2713933

[B42] Kan K. Schweizer T. A. Tam F. Graham S. J. (2012). Methodology for functional MRI of simulated driving: functional MRI of simulated driving. *Med. Phys.* 40:012301. 10.1118/1.4769107 23298106

[B43] Klauer S. G. Guo F. Simons-Morton B. G. Ouimet M. C. Lee S. E. Dingus T. A. (2014). Distracted driving and risk of road crashes among novice and experienced drivers. *New Engl. J. Med.* 370 54–59. 10.1056/nejmsa1204142 24382065 PMC4183154

[B44] Kline D. Kline T. Fozard J. Kosnik W. Schieber F. Sekuler R. (1992). Vision, aging, and driving: the problems of older drivers. *J. Gerontol.* 47 27–34. 10.1093/geronj/47.1.p27 1730855

[B45] Kok E. M. Jarodzka H. (2017). Before your very eyes: the value and limitations of eye tracking in medical education. *Med. Educ.* 51 114–122. 10.1111/medu.13066 27580633

[B46] Kramer A. F. Wiegmann D. A. Kirlik A. (2006). *AttentionFrom Theory to Practice.* Oxford: Oxford University Press.

[B47] Kret M. E. Sjak-Shie E. E. (2019). Preprocessing pupil size data: guidelines and code. *Behav. Res. Methods* 51 1336–1342. 10.3758/s13428-018-1075-y 29992408 PMC6538573

[B48] Künzle H. Akert K. Wurtz R. H. (1976). Projection of area 8 (frontal eye field) to superior colliculus in the monkey. an autoradiographic study. *Brain Res.* 117 487–492. 10.1016/0006-8993(76)90754-x 825196

[B49] LaBerge D. (2000). “Networks of attention,” in *The New Cognitive Neurosciences* Ed, Michael S. Gazzaniga. (Cambridge, MA: MIT Press) 711–724.

[B50] Land M. F. (2006). Eye movements and the control of actions in everyday life. *Prog. Retinal Eye Res.* 25 296–324. 10.1016/j.preteyeres.2006.01.002 16516530

[B51] Lee H. C. Cameron D. Lee A. H. (2003). Assessing the driving performance of older adult drivers: on-road versus simulated driving. *Accident Anal. Prevent.* 35 797–803. 10.1016/s0001-4575(02)00083-0 12850081

[B52] Lerner A. Bagic A. Hanakawa T. Boudreau E. A. Pagan F. Mari Z. (2009). Involvement of insula and cingulate cortices in control and suppression of natural urges. *Cereb. Cortex* 19 218–223. 10.1093/cercor/bhn074 18469316 PMC2638741

[B53] Lezak M. D. Howieson D. B. Loring D. W. Fischer J. S. (2004). *Neuropsychological Assessment.* New York, NY: Oxford University Press.

[B54] Li Y.-O. Eichele T. Calhoun V. D. Adali T. (2012). Group study of simulated driving fMRI data by multiset canonical correlation analysis. *J. Signal Process. Systems* 68 31–48. 10.1007/s11265-010-0572-8 23750290 PMC3673747

[B55] Liakakis G. Nickel J. Seitz R. J. (2011). Diversity of the inferior frontal gyrus—a meta-analysis of neuroimaging studies. *Behav. Brain Res.* 225 341–347. 10.1016/j.bbr.2011.06.022 21729721

[B56] Lundberg C. Johansson K. Ball K. Bjerre B. Blomqvist C. Braekhus A. (1997). Dementia and driving: an attempt at consensus. *Alzheimer Dis. Assoc. Disord.* 11 28–37. 10.1097/00002093-199703000-00006 9071442

[B57] Mack A. (2003). Inattentional blindness: looking without seeing. *Curr. Direct. Psychol. Sci.* 12 180–184. 10.1111/1467-8721.01256

[B58] Maffei A. Angrilli A. (2018). Spontaneous eye blink rate: an index of dopaminergic component of sustained attention and fatigue. *Int. J. Psychophysiol.* 123 58–63. 10.1016/j.ijpsycho.2017.11.009 29133149

[B59] Mangun G. R. Buonocore M. H. Girelli M. Jha A. P. (1998). ERP and fMRI measures of visual spatial selective attention. *Hum. Brain Mapp.* 6 383–389. 10.1002/(sici)1097-0193(1998)6:5/6<383::aid-hbm10>3.0.co;2-z 9788077 PMC6873372

[B60] Manor B. R. Gordon E. (2003). Defining the temporal threshold for ocular fixation in free-viewing visuocognitive tasks. *J. Neurosci. Methods* 128 85–93. 10.1016/s0165-0270(03)00151-1 12948551

[B61] Mansur A. Hird M. A. Desimone A. Pshonyak I. Schweizer T. A. Das S. (2018). Driving habits and behaviors of patients with brain tumors: a self-report, cognitive and driving simulation study. *Sci. Rep.* 15:4635. 10.1038/s41598-018-22937-y PMC5854700 29545606

[B62] Marandi R. Z. Gazerani P. (2019). Aging and eye tracking: in the quest for objective biomarkers. *Future Neurol.* 14:FNL33.

[B63] Marshall S. C. Gilbert N. (1999). Saskatchewan physicians’ attitudes and knowledge regarding assessment of medical fitness to drive. *CMAJ* 160 1701–1704. 10410630 PMC1230403

[B64] Matthys K. Smits M. Geest J. N. V. der, Lugt A. V. (2009). Mirror-Induced visual illusion of hand movements: a functional magnetic resonance imaging study. *Arch. Phys. Med. Rehabil.* 90 675–681. 10.1016/j.apmr.2008.09.571 19345786

[B65] Mazzone L. Yu S. Blair C. Gunter B. C. Wang Z. Marsh R. (2010). An fMRI study of frontostriatal circuits during the inhibition of eye blinking in persons with tourette syndrome. *Am. J. Psychiatry* 167 341–349. 10.1176/appi.ajp.2009.08121831 20080981 PMC4295823

[B66] McIntire L. K. McKinley R. A. Goodyear C. McIntire J. P. (2014). Detection of vigilance performance using eye blinks. *Appl. Ergon.* 45 354–362. 10.1016/j.apergo.2013.04.020 23722006

[B67] Moore T. Fallah M. (2001). Control of eye movements and spatial attention. *Proc. Natl. Acad. Sci. U S A.* 98 1273–1276. 11158629 10.1073/pnas.021549498 PMC14744

[B68] Moss H. E. Abdallah S. Fletcher P. Bright P. Pilgrim L. Acres K. (2005). Selecting among competing alternatives: selection and retrieval in the left inferior frontal gyrus. *Cereb. Cortex* 15 1723–1735. 10.1093/cercor/bhi049 15728742 PMC3838943

[B69] Murphy P. R. O’Connell R. G. O’Sullivan M. Robertson I. H. Balsters J. H. (2014). Pupil diameter covaries with bold activity in human locus coeruleus: pupil diameter and locus coeruleus activity. *Hum. Brain Mapp.* 35 4140–4154. 10.1002/hbm.22466 24510607 PMC6869043

[B70] National Motor Vehicle Crash Causation Survey (2008). *National Motor Vehicle Crash Causation Survey: Report to Congress, U.S. Department of Transportation National Highway Distracted Driving 2011. Traffic Safety facts U.S. Department of Transportation National Highway Traffic safety Association.* Washington, DC: National Motor Vehicle Crash Causation Survey.

[B71] Distracted Driving (2018). *Distracted Driving 2016. Traffic Safety Facts. U.S. Department of Transportation, National Highway Traffic Safety Association.* Washington, DC: National Highway Traffic Safety Association.

[B72] Neufang S. Akhrif A. Riedl V. Förstl H. Kurz A. Zimmer C. (2011). Disconnection of frontal and parietal areas contributes to impaired attention in very early Alzheimer’s disease. *J. Alzheimer’s Dis. JAD* 25 309–321. 10.3233/jad-2011-102154 21422523

[B73] Niezgoda M. Tarnowski A. Kruszewski M. Kamiński T. (2015). Towards testing auditory–vocal interfaces and detecting distraction while driving: a comparison of eye-movement measures in the assessment of cognitive workload. *Transportation Res. Part F: Traffic Psychol. Behav.* 32 23–34. 10.1016/j.trf.2015.04.012

[B74] Nij Bijvank J. A. Petzold A. Balk L. J. Tan H. S. Uitdehaag B. M. J. Theodorou M. (2018). A standardized protocol for quantification of saccadic eye movements: DEMoNS. *PLoS One* 13:e0200695. 10.1371/journal.pone.0200695 30011322 PMC6047815

[B75] Noyce D. A. Chitturi M. V. Nassereddine H. Santiago-Chaparro K. R. Bill A. R. (2017). *Neural Correlates of Older Driver Performance.* Washington, DC: University Transportation Center.

[B76] Nyström M. Andersson R. Holmqvist K. van de Weijer J. (2013). The influence of calibration method and eye physiology on eyetracking data quality. *Behav. Res. Methods* 45 272–288. 10.3758/s13428-012-0247-4 22956394

[B77] Ohira H. (1996). Eyeblink activity in a word-naming task as a function of semantic priming and cognitive load. *Percept. Mot. Skills* 82 835–842. 10.2466/pms.1996.82.3.835 8774018

[B78] Onitsuka T. Shenton M. E. Salisbury D. F. Dickey C. C. Kasai K. Toner S. K. (2004). Middle and inferior temporal gyrus gray matter volume abnormalities in chronic schizophrenia: an MRI study. *Am. J. Psychiatry* 161 1603–1611. 10.1176/appi.ajp.161.9.1603 15337650 PMC2793337

[B79] Orr C. Hester R. (2012). Error-related anterior cingulate cortex activity and the prediction of conscious error awareness. *Front. Hum. Neurosci.* 6:177. 10.3389/fnhum.2012.00177 22723775 PMC3377932

[B80] Ortiz N. Ramnarayan M. Mizenko K. (2016). The effect of distraction on road user behavior: an observational pilot study across intersections in Washington, DC. *J. Transport Health* 3:S67.

[B81] Palinko O. Kun A. L. Shyrokov A. Heeman P. (2010). “Estimating cognitive load using remote eye tracking in a driving simulator,” in *Proceedings of the 2010 Symposium on Eye-Tracking Research & Applications*, (New York, NY: ACM), 141–144.

[B82] Pardo J. V. Fox P. T. Raichle M. E. (1991). Localization of a human system for sustained attention by positron emission tomography. *Nature* 349 61–64. 10.1038/349061a0 1985266

[B83] Paus T. (2001). Primate anterior cingulate cortex: where motor control, drive and cognition interface. *Nat. Rev. Neurosci.* 2 417–424. 10.1038/35077500 11389475

[B84] Pavisic I. M. Firth N. C. Parsons S. Rego D. M. Shakespeare T. J. Yong K. X. X. (2017). Eyetracking metrics in young onset Alzheimer’s disease: a window into cognitive visual functions. *Front. Neurol.* 8:377. 10.3389/fneur.2017.00377 28824534 PMC5545969

[B85] Petersen S. E. Fox P. T. Posner M. I. Mintun M. Raichle M. E. (1989). Positron emission tomographic studies of the processing of singe words. *J. Cogn. Neurosci.* 1 153–170. 10.1162/jocn.1989.1.2.153 23968463

[B86] Raichle M. E. (2015). The brain’s default mode network. *Annu. Rev. Neurosci.* 38 433–447. 25938726 10.1146/annurev-neuro-071013-014030

[B87] Recarte M. A. Nunes L. M. (2003). Mental workload while driving: effects on visual search, discrimination, and decision making. *J. Exp. Psychol. Appl.* 9 119–137. 10.1037/1076-898x.9.2.119 12877271

[B88] Renier L. A. Anurova I. De Volder A. G. Carlson S. VanMeter J. Rauschecker J. P. (2010). Preserved functional specialization for spatial processing in the middle occipital gyrus of the early blind. *Neuron* 68 138–148. 10.1016/j.neuron.2010.09.021 20920797 PMC2951740

[B89] Rizzo M. (2011). Impaired driving from medical conditions: a 70-year-old man trying to decide if he should continue driving. *JAMA* 305:1018. 10.1001/jama.2011.252 21364126 PMC3289256

[B90] Rueckert L. Grafman J. (1996). Sustained attention deficits in patients with right frontal lesions. *Neuropsychologia* 34 953–963. 10.1016/0028-3932(96)00016-4 8843061

[B91] Rueckert L. Grafman J. (1998). Sustained attention deficits in patients with lesions of posterior cortex. *Neuropsychologia* 36 653–660. 10.1016/s0028-3932(97)00150-4 9723936

[B92] Schneider W. X. (1995). VAM: a neuro-cognitive model for visual attention control of segmentation, object recognition, and space-based motor action. *Visual Cogn.* 2 331–376. 10.1080/13506289508401737

[B93] Schweizer T. A. Kan K. Hung Y. Tam F. Naglie G. Graham S. J. (2013). Brain activity during driving with distraction: an immersive fMRI study. *Front. Hum. Neurosci.* 7:53. 10.3389/fnhum.2013.00053 23450757 PMC3584251

[B94] Siegle G. J. Steinhauer S. R. Stenger V. A. Konecky R. Carter C. S. (2003). Use of concurrent pupil dilation assessment to inform interpretation and analysis of fMRI data. *NeuroImage* 20 114–124. 10.1016/s1053-8119(03)00298-2 14527574

[B95] Spiers H. J. Maguire E. A. (2007). Neural substrates of driving behaviour. *NeuroImage* 36 245–255. 10.1016/j.neuroimage.2007.02.032 17412611 PMC2570440

[B96] Stinchcombe A. Gagnon S. (2013). Aging and driving in a complex world: exploring age differences in attentional demand while driving. *Transportation Res. Part F: Traffic Psychol. Behav.* 17 125–133. 10.1016/j.trf.2012.11.002

[B97] Strayer D. L. Drew F. A. (2004). Profiles in driver distraction: effects of cell phone conversations on younger and older drivers. *Hum. Factors: J. Hum. Factors Ergonom. Soc.* 46 640–649. 10.1518/hfes.46.4.640.56806 15709326

[B98] Swick D. Ashley V. Turken A. U. (2008). Left inferior frontal gyrus is critical for response inhibition. *BMC Neurosci.* 9:102. 10.1186/1471-2202-9-102 18939997 PMC2588614

[B99] Talairach J. Tournoux P. (1988). *Co-planar Stereotaxic Atlas of the Human Brain: 3-Dimensional Proportional System: an Approach to Cerebral Imaging.* New York, NY: Thieme Medical Publishers, Inc.

[B100] Tao L. Wang Q. Liu D. Wang J. Zhu Z. Feng L. (2020). Eye tracking metrics to screen and assess cognitive impairment in patients with neurological disorders. *Neurol. Sci.* 41 1697–1704. 10.1007/s10072-020-04310-y 32125540

[B101] Thompson K. R. Johnson A. M. Emerson J. L. Dawson J. D. Boer E. R. Rizzo M. (2012). Distracted driving in elderly and middle-aged drivers. *Accident Anal. Prevent.* 45 711–717. 10.1016/j.aap.2011.09.040 22269561 PMC3266512

[B102] Tops M. Boksem M. A. S. (2011). A potential role of the inferior frontal gyrus and anterior insula in cognitive control, brain rhythms, and event-related potentials. *Front. Psychol.* 2:330. 10.3389/fpsyg.2011.00330 22084637 PMC3212750

[B103] Tranel D. Damasio H. Damasio A. R. (1997). A neural basis for the retrieval of conceptual knowledge. *Neuropsychologia* 35 1319–1327. 10.1016/s0028-3932(97)00085-7 9347478

[B104] Traffic safety Association. (2013). *Distracted Driving 2011. Traffic Safety Facts*. Fraser, MI: Traffic safety Association.

[B105] Tsai Y.-F. Viirre E. Strychacz C. Chase B. Jung T.-P. (2007). Task performance and eye activity: predicting behavior relating to cognitive workload. *Aviat. Space Environ. Med.* 78 B176–B185. 17547318

[B106] Uchiyama Y. Ebe K. Kozato A. Okada T. Sadato N. (2003). The neural substrates of driving at a safe distance: a functional MRI study. *Neurosci. Lett.* 352 199–202. 10.1016/j.neulet.2003.08.072 14625019

[B107] Uchiyama Y. Toyoda H. Sakai H. Shin D. Ebe K. Sadato N. (2012). Suppression of brain activity related to a car-following task with an auditory task: an fMRI study. *Transportation Res. Part F: Traffic Psychol. Behav.* 15 25–37. 10.1016/j.trf.2011.11.002

[B109] Van Orden K. F. Limbert W. Makeig S. Jung T.-P. (2001). Eye activity correlates of workload during a visuospatial memory task. *Hum. Fact.* 43 111–121. 10.1518/001872001775992570 11474756

[B110] Vernet M. Quentin R. Chanes L. Mitsumasu A. Valero-Cabré A. (2014). Frontal eye field, where art thou? anatomy, function, and non-invasive manipulation of frontal regions involved in eye movements and associated cognitive operations. *Front. Integr. Neurosci.* 8:66. 10.3389/fnint.2014.00066 25202241 PMC4141567

[B111] Wandell B. A. Dumoulin S. O. Brewer A. A. (2007). Visual field maps in human cortex. *Neuron* 56 366–383. 10.1016/j.neuron.2007.10.012 17964252

[B112] Ware M. Feng J. Nam C. S. (2020). “Neuroergonomics behind the wheel: neural correlates of driving,” in *Neuroergonomics: Principles and Practice*, ed. Nam C. S. (Cham: Springer International Publishing), 353–388. 10.1007/978-3-030-34784-0_18

[B113] Weissman D. H. Prado J. (2012). Heightened activity in a key region of the ventral attention network is linked to reduced activity in a key region of the dorsal attention network during unexpected shifts of covert visual spatial attention. *NeuroImage* 61 798–804. 10.1016/j.neuroimage.2012.03.032 22445785

[B114] Wilkins A. J. Shallice T. McCarthy R. (1987). Frontal lesions and sustained attention. *Neuropsychologia* 25 359–365. 10.1016/0028-3932(87)90024-8 3601041

[B115] Withaar F. K. Brouwer W. H. Van Zomeren A. H. (2000). Fitness to drive in older drivers with cognitive impairment. *J. Int. Neuropsychol. Soc.* 6 480–490. 10.1017/s1355617700644065 10902417

[B116] Wu D. Huang H. Liu N. Miao D. (2019). Information processing under high and low distractions using eye tracking. *Cogn. Process.* 20 11–18. 10.1007/s10339-018-0876-3 30051237

[B117] Zekveld A. A. Heslenfeld D. J. Johnsrude I. S. Versfeld N. J. Kramer S. E. (2014). The eye as a window to the listening brain: neural correlates of pupil size as a measure of cognitive listening load. *NeuroImage* 101 76–86. 10.1016/j.neuroimage.2014.06.069 24999040

